# Social and policy interventions to reduce hospital admissions among socioeconomically disadvantaged groups in OECD countries with universal health care: a systematic review

**DOI:** 10.1136/bmjph-2025-002592

**Published:** 2025-09-23

**Authors:** Behrouz Nezafat Maldonado, William Bell, Jasmine Olivera, Fiona Beyer, Mark Lambert, Richard Thomson, Richard Cookson, Clare Bambra, Sarah Sowden

**Affiliations:** 1 School of Public Health, Imperial College London, London, UK; 2 Population Health Sciences Institute, Newcastle University, Newcastle upon Tyne, UK; 3 Independent, New York, New York, USA; 4 NHS England, Newcastle upon Tyne, UK; 5 Centre for Health Economics, University of York, York, UK

**Keywords:** Public Health, Preventive Medicine, Sociodemographic Factors

## Abstract

**Objectives:**

Socioeconomic disadvantage increases the risk of acute illnesses and injuries requiring hospital admission, some of which are avoidable. This systematic review aimed to identify the impact of interventions on hospital admissions in socioeconomically disadvantaged populations and identify knowledge gaps.

**Design:**

Systematic review (PROSPERO, CRD42019153666).

**Data sources:**

We searched MEDLINE (OVID), Embase (OVID), CINAHL (EBSCO), Cochrane CENTRAL (Wiley) and the Web of Knowledge platforms.

**Eligibility criteria:**

Studies published between 1 January 2000 and 1 April 2024. We included quantitative studies that included a socioeconomically disadvantaged population, conducted studies in countries members of the Organisation for Economic Co-operation and Development (OECD) with universal healthcare and reported on hospital admission or readmissions.

**Data extraction and synthesis:**

We assessed study quality using the Effective Public Health Practice Project tool. We summarised studies using a narrative synthesis approach and present findings using vote counting as a measure of effect.

**Results:**

We included 20 studies of interventions targeted towards socioeconomically disadvantaged populations. Their impacts on hospitalisations of interventions, grouped under three domains—(1) population level health and social policy, (2) health and care service-based interventions and (3) integrative interventions—were mixed. Through vote counting, we found some evidence that social policy interventions targeting socioeconomically disadvantaged groups have an important impact on hospitalisations, especially those focused on improved housing and income.

**Conclusions:**

While ongoing efforts to ensure that healthcare interventions improve the equity of access, experience and outcome are warranted, social policy interventions that address the wider determinants of health, such as housing, income and education, hold promise for controlling rates of hospital admissions in socioeconomically disadvantaged groups. This underscores the value of multi-sectoral action to reduce inequalities. Future studies should explore the long-term outcomes of interventions, particularly integrative ones, which may bring benefits in the long term but not so much in the short term.

**PROSPERO registration number:**

CRD42019153666

**WHAT IS ALREADY KNOWN ON THIS TOPIC**

- People from socioeconomically disadvantaged backgrounds are at a higher risk of unplanned and preventable hospitalisations. There is a lack of robust evidence regarding the effectiveness of interventions to reduce these hospitalisations.

**WHAT THIS STUDY ADDS**

- Few studies have reported the impact of population health and policy interventions, health and care service interventions and integrative interventions on hospitalisations among socioeconomically disadvantaged groups, and intervention impacts are mixed. Social policy interventions that improve income and housing reduce hospital admissions. However, findings should be interpreted with caution due to the heterogeneity of studies. Future studies should explore the long-term outcomes of integrative interventions.

**HOW THIS STUDY MIGHT AFFECT RESEARCH, PRACTICE OR POLICY**

- Investment in concerted action to address the wider determinants of health such as housing, income and health education through population policy and integrative interventions, alongside promoting the equity of access, experience and outcome from healthcare, appears important to reduce hospitalisation rates in populations experiencing socioeconomic disadvantage.

## Background

 Healthcare expenditure is increasing globally, underpinned by the rapidly ageing population, healthcare staff shortages, chronic conditions as well as external shocks such as inflation and climate change that raise costs.^1 2^ To promote the fiscal sustainability of health systems, policy makers aim to shift expenditure from reactive, treatment-focused hospital-based models to healthcare approaches that prioritise prevention and health promotion in primary care or community settings.^3 4^ This can be achieved through policies that foster healthier populations, minimise ineffective spending and reduce hospital admissions.^5^

Socioeconomically disadvantaged groups are disproportionately affected by the rising cost of living, which negatively impacts their health outcomes.^6^ These groups often have poorer access to healthcare and outcomes from preventive and planned healthcare, which further impacts their health.^7^ Population-based research has shown that adults and children living in deprived areas use more emergency care and experience higher rates of hospital admissions compared with those in affluent areas.^8^,^10^ Interventions that target this particular population to meet their health needs may reduce acute admissions to hospital.

Previous research suggests that accessible, multisectoral, locally designed and well-resourced interventions that target disadvantaged communities may bring about positive health outcomes.^11 12^ However, interventions requiring a high level of self-agency may exacerbate inequalities.^1113^,^15^

Universal health systems provide access to high-quality care for the entire population, regardless of socioeconomic status.^16^ In such systems, hospital admissions are generally not associated with out-of-pocket payment, meaning that the full cost of the admissions is covered by the system. Therefore, it is especially important in universal health systems to reduce acute hospital admissions among socioeconomically disadvantaged groups through interventions that improve their overall health. Interventions may not only improve health and prevent the development of new conditions but also reduce the severity of existing ones (eg, asthma exacerbations) and prevent hospital admissions associated with environmental factors, such as injuries or exposure to air pollution. Consequently, such interventions may reduce hospital admissions in both the short and long terms.

This systematic review explores the impact of interventions, aimed at socioeconomically disadvantaged populations, on hospital admissions in OECD countries with universal healthcare.

## Methods

We carried out a systematic review of studies, including grey literature, published between 1 January 2000 and 1 April 2024. Focusing on the last 24 years ensures that findings are relevant and encompass pre-pandemic as well as post-pandemic developments and shifts in population health.

We followed Preferred Reporting Items for Systematic Reviews and Meta-Analysis-Equity (PRISMA-E) and Synthesis Without Meta-analysis (SWiM) in systematic reviews: reporting guideline and Cochrane Handbook for Systematic Reviews of Interventions guidance.^17^,^20^ The protocol was registered on the International Prospective Register of Systematic Reviews (PROSPERO, CRD42019153666) and was previously published in a peer-reviewed journal.^21^

### Search strategy and selection criteria

We searched six electronic databases: MEDLINE (Ovid), Embase (Ovid), CINAHL (EBSCO), Cochrane CENTRAL (Wiley), Science Citation Index (Web of Science) and Social Science Citation Index (Web of Science). The search strategy was designed in MEDLINE to find interventions to reduce hospitalisations and used a validated filter for inequalities.^22^ A complete search strategy for MEDLINE is provided in online supplemental file 1. References of included studies were also screened to identify additional papers, and expert collaborators of the UNderstanding Factors that explain Avoidable hospital admission Inequalities Research (UNFAIR) study (http://bit.ly/UNFAIRstudy) were contacted to provide relevant references (online supplemental file 2). In addition, we searched key websites (Health Foundation, Nuffield Trust, OECD, WHO, EuroStat and King’s Fund) for relevant grey literature.

### Inclusion and exclusion criteria

We included interventions implemented either across different groups or using pre- and post-measures of the outcome of interest. The primary outcome of interest was admissions to hospitals. Our population of interest was socioeconomically disadvantaged groups, as defined by the PROGRESS-Plus framework (table 1).^23^ The PROGRESS-Plus framework provides an acronym—place of residence, race/ethnicity/culture/language, occupation, gender/sex, religion, education, socioeconomic status and social capital (‘PROGRESS’)—to assess the effects of interventions through an equity lens. We only focus on low socioeconomic groups and do not consider other factors that may lead to inequalities such as race/ethnicity. We include all countries with the membership of the OECD at the time of the study and exclude those OECD countries without a universal public healthcare (UHC). For example, the United States and Switzerland do not have UHC covering over 80% of their population through autonomic or compulsory insurance coverage, so we excluded them and other non-UHU countries as it would be difficult to generalise findings.^24^ Papers which examined the differential effectiveness of interventions implemented across socioeconomic groups, as opposed to being targeted in their implementation solely at socioeconomically disadvantaged groups, have been excluded from this review. Systematic reviews were not included, but their references were hand-searched to identify additional papers.

**Table 1:** Definition of socioeconomically disadvantaged groups using relevant PROGRESS-PLUS factors

	Example population
Place of residence	Inner-city urban, people experiencing homelessness
Occupation	Unemployed persons
Education	Low health literacy
Socioeconomic status	Area deprivation, low-income population

Records found in any language, targeting socioeconomically disadvantaged groups (table 1), and designed as randomised controlled trials, cohort studies, case–control studies or quasi-experimental studies were included.

We used EPPI-Reviewer 4 software to deduplicate studies and carry out the screening.^25^ Two reviewers (BNM and SS) independently screened titles and abstracts against the inclusion criteria. 10% of papers were double-screened independently and then reviewed to ensure a consistent approach. Full texts of studies were screened by both reviewers independently, against the inclusion criteria. Disagreements at any stage were resolved through consultation with a third reviewer (FB).

### Interventions of interest

We planned to categorise the interventions into four domains: population health and public policy interventions, health and care service-based interventions, community-based interventions and integrative interventions. However, in a deviation to the published protocol, we found that integrative interventions were community-based, so we collapsed community-based interventions into the integrative intervention domain and classified according to these three domains (box 1). Categorisation of the interventions was agreed by authors carrying out data extraction (BNM, JO and WB), and any disagreements were resolved through consultation with a third author (SS).

Box 1 **Domains of actions of interventions (modified from protocol)**

Population health and policy interventions included legal, fiscal, structural, organisational and policy changes that aimed to modify health-related behaviours or social, commercial or economic determinants of health. Examples of such interventions would include sugar taxation to change health behaviours.

Health and care service-based interventions include those that are carried out within a healthcare or a social care setting, such as hospital-at-home initiatives or the expansion of primary care.

Integrative interventions are those implemented across primary and secondary care and community services as well as non-healthcare organisations. These interventions may be implemented at local, regional or national level and are driven by professionals working directly with service users as supposed to being implemented by policy makers.

We presented a proposed framework in the protocol, as specified in PRISMA-E guidelines, and outlined the characteristics and interrelationships between these domains.^21^

### Data extraction and quality assessment

Data for included studies were extracted by three reviewers (BNM, JO and WB) using a bespoke form within EPPI-Reviewer 4.

Study quality and risk of bias were assessed using the Effective Public Health Practice Project (EPHPP) tool.^26^ The EPHPP tool applies to a range of quantitative study designs and allowed us to critically appraise all the study designs in our review, but it was not used to guide the inclusion of studies. Studies were assessed by two reviewers (BNM and JO), and any disagreements in quality assessment judgements were solved through discussion between the reviewers and consultation with the wider team if needed (FB and SS). We report according to PRISMA and SWiM guidelines (online supplemental files 3 and 4).

### Deviations from the protocol

The level of implementation information contained within the papers was insufficient to use the Template for Intervention Description and Replication for Population Health and Policy to extract relevant contextual information, a step that we outlined in the systematic review protocol as something we could undertake.

Although in the protocol we present four domains of actions for interventions, we collapsed the community domain and the integrative domain into one, as we found that all integrative interventions were also community-based and bridged the gap between hospital-based care and community care.

Due to the vast number of records identified in the search, we have split the systematic review into two based on whether interventions were targeting a specific socioeconomically disadvantaged group or applied to a whole population but where the differential effectiveness of the intervention across socioeconomic groups had been examined. This is a deviation from the protocol that was agreed on to ensure synthesis and interpreting results were achievable. The review evaluating the differential effectiveness of interventions on hospital admissions across socioeconomic groups has been published.^27^

### Data synthesis

A meta-analysis was not possible due to study heterogeneity and variability in outcome measurement. Thus, we report a narrative synthesis together with descriptive vote counting on the direction of effect, following the Cochrane Handbook for Systematic Reviews of Interventions guidance.^19^ Vote counting compares the number of effects showing benefit to the number of effects showing harm for a particular outcome without taking into account subjective decisions or statistical significance. We categorise each intervention as showing an increase or a decrease or no impact on hospitalisations based on the observed direction of effect alone. Following this, a count of the number of effects is created and compared. Neither statistical significance nor the size of the effect is considered, and so, this method does not account for differences in relative sizes of the studies.

The narrative synthesis is presented according to the domain and the population-based clusters of interest, for example, people experiencing homelessness (PEH) as defined by authors. Interventions that included multiple population groups were included in every relevant cluster, so the reported clusters are not mutually exclusive.

We report on admissions to hospital, hospitalisations, as described in included studies—generally, this is reported as being admitted to hospital regardless of the duration of the stay. Vote counting was used to compare the direction of the effect of each intervention on the outcomes of interest, showing those interventions that increased hospitalisations or readmissions compared with those that decreased the outcome without quantification of the effect.

The funder of the study had no role in study design, data collection, data analysis, data interpretation, writing of the report or publication process.

## Results

**Figure 1:**
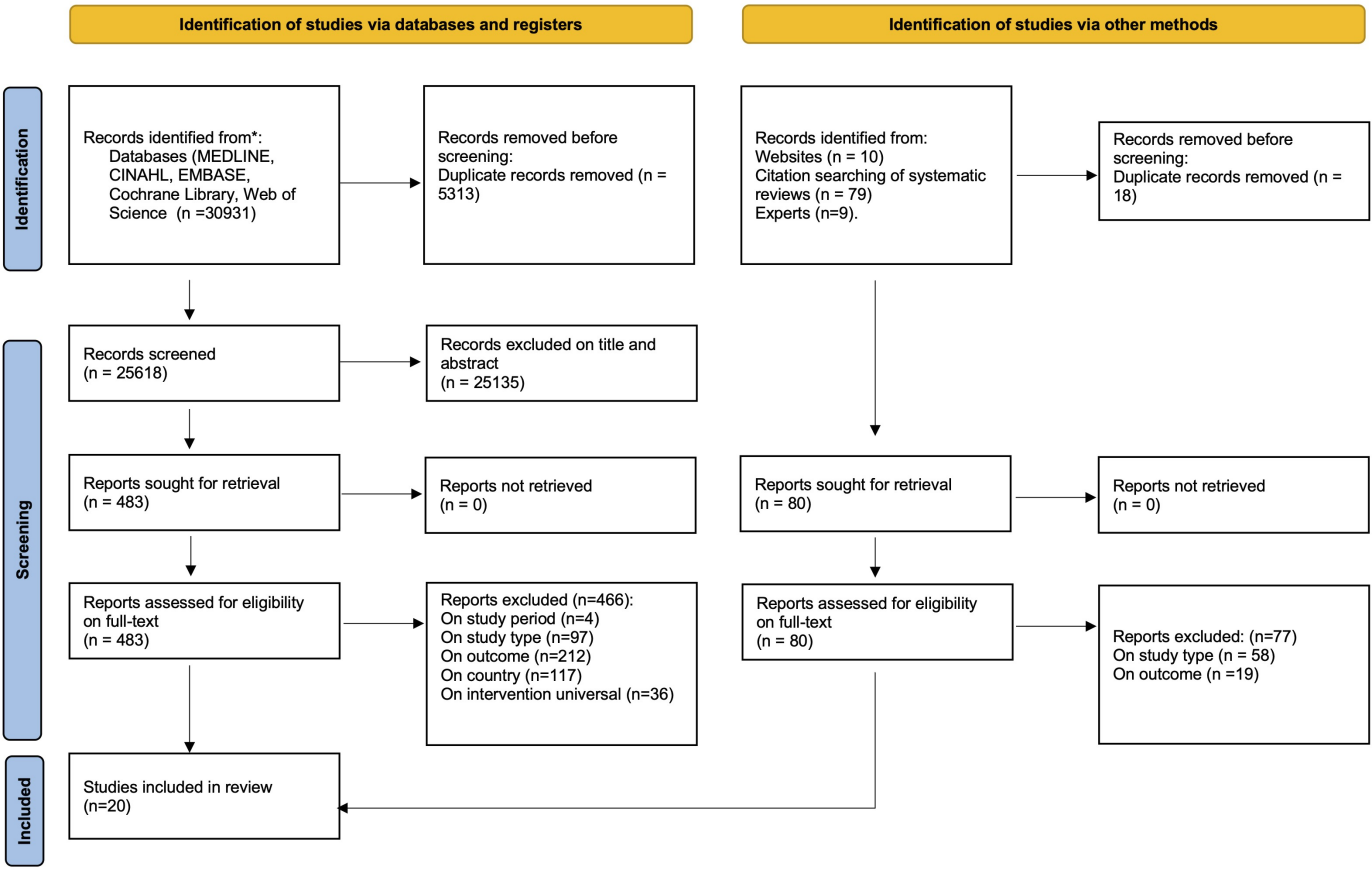
PRISMA flow diagram.

Our research identified 30 931 records from the initial database search and 128 through additional methods. After removing duplicates, titles and abstracts of 25 618 studies were screened (figure 1). 563 full texts were reviewed, and 20 studies were included in the final review. No studies published in a non-English language met the inclusion criteria. Many studies were excluded as they did not target a socioeconomically disadvantaged group or report on the intervention effect by socioeconomic status and hospitalisation rates. The reasons for exclusion at the full-text review stage are available in online supplemental file 5.

We include interventions from several countries: four studies from the United Kingdom,^28^,^31^ three from Canada,^32^,^34^ two from Australia,^35^,^38^ two from Germany,^39 40^ two from New Zealand^41 42^ and one each from France,^43^ Israel,^44^ Italy,^45^ Spain,^46^ South Korea^47^ and Turkey^48^ and a multicentre international study in Italy, Germany, the Netherlands and the United Kingdom.^49^ Switzerland and Bulgaria were also included in the multicentre international study. However, the results from these were not included in the analysis for reasons described previously. Study designs varied and included randomised control trials, cohort studies, cross-sectional time series studies and quasi-experimental studies. The summary characteristics of the included studies are found in table 2. Further details on the interventions evaluated are available in online supplemental file 6.

**Table 2:** Study characteristics

Author (year)	Country	Study type	Study duration	Population	Definition of hospitalisation	Unit of analysis	Domain of action	Intervention summary
Adesanya^35^ (2005)	Australia	Cross-sectional time series	18 months	Unemployed persons	Admission to adult inpatient unit	Individual patients	Health and care service-based	Crisis assessment and treatment services
Burns *et al*^49^ (2007)	United Kingdom, Germany, Italy, Switzerland, Netherlands and Bulgaria,	Randomised control trial	18 months	Unemployed persons	Undefined	Individual patients	Population health and policy	Individualised job placement and support programmes
Castriotta *et al*^45^ (2020)	Italy	Cohort study	8 years	Low-income population	Admissions to regional hospitals: ordinary (planned+day hospital) and urgent	Individual residents	Integrative	Habitat Microaree: the community-based intervention consisted of facilitating access to social services and outpatient healthcare facilities, coordinating intersectoral public services and specifically planning hospital discharge
Downing *et al*^28^ (2020)	United Kingdom	Cohort study	5 years pre and post	Low-income population	Emergency admission for cardiovascular disease	Regional	Integrative	Consultant-led, community-based cardiovascular diagnostic, treatment and rehabilitation service
Forget^32^ (2011)	Canada	Quasi-experimental	2 years	Low-income population	Undefined	Regional	Population health and policy	Guaranteed annual income for all families, on a sliding scale dependent on alternative income and social funding
Gazey *et al*^36^ (2018)	Australia	Cross-sectional time series	12 months pre and post	People experiencing homelessness	Undefined	Individual residents	Integrative	Medical respite care in a home-like environment for homeless people post discharge
Horwitz *et al*^44^ (2021)	Israel	Prospective study	12 months	Low-income population	Undefined	Individual patients	Integrative	Health education on asthma, acknowledgement and elimination of risk factors and discussing possible treatment strategies during an educational meeting followed by personalised plan, a home visit and two telephone sessions at 6 and 12 months
Hwang *et al*^33^ (2011)	Canada	Quasi-experimental	18 months	People experiencing homelessness	Undefined	Individual residents	Integrative	Supportive housing, defined as subsidised housing in conjunction with site-based social services (and on-site free medical and dental care)
Jackson *et al*^42^ (2011)	New Zealand	Cross-sectional time series	10 years	Low-income population	Undefined	Individual residents	Integrative	Funded housing quality improvements and health education interventions
Kackin and Kahraman^48^ (2020)	Turkey	Quasi-experimental study	3 months	Low-income population	Emergency hospital admission for asthma	Individual patients	Health and care service-based	In-person discharge training on basics of asthma, triggering factors, symptom control and hygiene followed by home monitoring visits 3 months post-discharge to assess compliance with treatment
Kim and Shon^47^ (2018)	South Korea	Quasi-experimental study	3 years	Low-income population	Preventable hospitalisation	National level	Population health and policy	Medical aid health services to increase population level coverage
Lichtl and Bozorgmehr^39^ (2019)	Germany	Cross-sectional time series	3 years	Low-income population	Undefined	Regional level	Health and care service-based	Walk-in clinic to improve access to healthcare for asylum seekers
Lopez Cabezas *et al*^46^ (2006)	Spain	Randomised control trial	12 months	Low-income population	Heart failure hospitalisation	Individual patient	Health and care service-based	Heart failure education and follow-up from a pharmacist
Rodgers *et al*^29^ (2018)	United Kingdom	Cross-sectional time series	10 years and 3 months	Low-income population	Undefined	Regional	Population health and policy	House quality improvements in parts not up to national quality level
Tinland *et al*^43^ (2020)	France	Randomised control trial	24 months	People experiencing homelessness	Undefined	Individual patient	Integrative	Independent housing with mental health support teams with a recovery-orientated approach—Housing First programme
Goldzahl *et al*^30^ (2022)	United Kingdom	Cross-sectional retrospective study	3 years	Low-income population	Undefined	Regional	Integrative	MDGs of multi-professionals sharing information, discussing and care planning for high-risk patient cases. MDGs increased support and support to community services. An integrated contact centre providing advice to navigate health and social care services
Malden *et al*^31^ (2023)	United Kingdom	Cross-sectional time series	12 months pre and post	People experiencing homelessness	Undefined	Individual patients	Integrative	Hospital-in-reach programme— multicomponent intervention depending on needs to support safe accommodation, income maximisation and community support after discharge
Norris *et al*^41^ (2023)	New Zealand	Randomised controlled trial	1 year	Low-income population	Undefined	Individual patients	Population health and policy	Exception from prescription co-payments
Ress and Wild^40^ (2024)	Germany	Cross-sectional time series	24 months pre and post	Low-income population	Undefined	Regional	Integrative	Health, social and community care providers coordinate the access to services including social prescribing and provide health education
Salvalaggio *et al*^34^ (2022)	Canada	Cross-sectional time series	180 days prior to and 360 days post intervention	Low-income population	Undefined	Individual patient	Health and care service-based	Multidisciplinary team with expertise in addiction medicine to promote continuity of care between hospital and community care

MDG, multi-disciplinary group .

While very few studies were identified in the literature, interventions aimed at reducing hospital admissions in socioeconomically disadvantaged populations were found across all three domains of activity. Five studies presented population health and policy-level interventions, five studies reported health and care service-based interventions and 10 studies presented integrative interventions. Only one study involved service users in the planning, design, implementation and evaluation of the interventions. We present the overall direction of the effect on hospitalisation for included studies (table 3). Table 3 demonstrates that the impact of interventions on hospitalisations in socioeconomically disadvantaged groups was mixed. For the population health and public policy domain, 4 out of 5 studies indicated decreasing hospitalisations associated with an intervention with one study reporting increases in hospitalisations following the intervention. However, this was an intentional and expected increase in healthcare utilisation through planned pathways to increase access to healthcare and address unmet needs. For the health and care service-based domain, two studies showed that interventions decreased hospitalisations, whereas the remaining three indicated no change in hospitalisations on account of the interventions. For the integrative domain, five studies reported decreasing hospitalisations, one reported increasing hospitalisation and four reported no impact of the intervention on hospitalisations.

**Table 3:** Direction of effect on hospitalisation for each domain

	Population group	Quality assessment	Direction of effect on hospitalisation (increasing, decreasing and no impact)
Population health and policy interventions			
Kim and Shon^47^ (2018)	Low-income population	STRONG	↑*
Rodgers *et al*^29^ (2018)	People experiencing homelessness	STRONG	↓
Burns *et al*^49^ (2007)	Unemployed persons	MODERATE	↓
Forget^32^ (2011)	Low-income population	WEAK	↓
Norris *et al*^41^ (2023)	Low-income population	STRONG	↓
Health and care service-based interventions			
Lichtl and Bozorgmehr^39^ (2019)	Low-income population	STRONG	↔
Lopez Cabezas *et al*^46^ (2006)	Low-income population	STRONG	↓
Adesanya^35^ (2005)	Unemployed persons	MODERATE	↔
Kackin and Kahraman^48^ (2020)	Low-income population	WEAK	↓
Salvalaggio *et al*^34^ (2022)	Low-income population	WEAK	↔
Integrative interventions			
Malden *et al*^31^ (2023)	People experiencing homelessness	MODERATE	↓
Jackson *et al*^42^ (2011)	People experiencing homelessness	STRONG	↓
Gazey *et al*^36^ (2018)	People experiencing homelessness	STRONG	↓
Tinland *et al*^43^ (2020)	People experiencing homelessness	STRONG	↔
Castriotta *et al*^45^ (2020)	Low-income population	MODERATE	↓
Downing *et al*^28^ (2019)	Low-income population	MODERATE	↓
Hwang *et al*^33^ (2011)	People experiencing homelessness	MODERATE	↔
Horwitz *et al*^44^ (2021)	Low-income population	WEAK	↔
Goldzahl *et al*^30^ (2022)	Low-income population	MODERATE	↔
Ress and Wild^40^ (2024)	Low-income population	MODERATE	↑

Quality ratings: STRONG (no WEAK ratings), MODERATE (one WEAK rating) and WEAK (two or more WEAK ratings).

Direction of effect: ↑, increasing hospitalisations; ↔, no change in hospitalisations; ↓, decreasing hospitalisations.

* This intervention set out planned pathways to increase access to healthcare, and therefore, the authors believe that an increase in planned and appropriate hospitalisations should not be considered a negative outcome.

We carried out a quality assessment for each study, and most studies were rated as strong or moderate quality (table 3). Participant selection, the study design used and the lack of randomisation or blinding played a key role in those studies rated low quality. The global quality rating is summarised in table 3. There was no apparent association between the quality of the study and the study findings.

### Population health and policy interventions

Five studies implemented interventions focused on population health and policy. Of these, four studies were carried out in a single country—namely, Canada, New Zealand, South Korea and the United Kingdom—and one was a multicentre study across Germany, Italy, the Netherlands and the United Kingdom, among other countries that did not meet the inclusion criteria for this study.

#### Unemployed persons

We found one study evaluating policy changes that aimed to improve health among unemployed persons with concurrent severe mental health illness. This multicentre international randomised controlled trial aimed to assess the effectiveness of individual placement and support (IPS) programmes across six European countries compared with traditional vocational services.^49^ The trial was rated as moderate quality due to high drop-rate in recruitment and follow-up. The IPS is a policy intervention that consisted of individualised job placements and support programme for service users, an intervention that was compared with vocational service. The trial found that individuals in vocational service were more likely to be re-admitted (31% risk of re-admission) to the hospital than those assigned to IPS (20% risk of readmission)—a difference of –11·2% (–21·5 to –0·90).

#### Low-income population

Four interventions focused on deprived or low-income urban populations. One study in the United Kingdom hypothesised that improving housing and housing environment may lead to better health. Improving housing may reduce admissions to hospital in the following ways: first, admissions for fall injuries caused by slipping on uneven steps or having no handrails or trailing extension leads may be reduced through upgrades to kitchens, bathrooms, electrical systems and garden paths.

Second, improving insulation and heating improves thermal efficiency and weather proofing, which in turn has an impact on damp, mould and cold in the house, which may lead to a reduction in hospital admissions for exacerbations of pre-existing respiratory and circulatory conditions. Finally, refitting kitchens and bathrooms, improving heating systems and insulation, windows and doors, may reduce concerns about crime and antisocial behaviour and concerns relating to fuel poverty, damp and mould, which may ease levels of stress, anxiety and depression, which may potentially have led to mental health hospital admissions. The study, rated as strong quality, reported that housing improvements reduced hospitalisations through cross-sectional time-series analyses. Rodgers *et al* carried out housing improvements to meet national quality standards across social housing in Wales, UK, for low-income populations.^29^ They reported that improving electrical systems led to 39% fewer hospital admissions compared with those living in homes in which they were not part of the intervention (incidence rate ratio (IRR): 0.61, 95% CI: 0.53 to 0.72; p<0.01). Reduced admissions were also associated with improvements in windows and doors (IRR: 0.71, 95% CI: 0.63 to 0.81; p<0.01), wall insulation (IRR: 0.75, 95% CI: 0.67 to 0.84; p<0.01) and gardens and estates (IRR: 0.73, 95% CI: 0.64 to 0.83; p<0.01) for those living in homes in which these improvements were carried out.

Three studies aimed to improve access to health services by increasing purchasing power. One study in Canada increased the family’s income through a guaranteed annual income, one in New Zealand abolished prescription co-payments/charges and a third one in South Korea increased healthcare coverage.^32 41 47^ The findings across these interventions showed that increasing families’ income through a guaranteed annual income or by reducing the cost of prescription drugs led to a reduction in hospitalisation. On the other hand, increasing access to healthcare through medical aid programmes led to an increase in hospital admissions but through planned pathways.

A quasi-experimental retrospective study of the Manitoba Basic Annual Income Experiment from 1974 to 1979 found an overall reduction in hospitalisations in those receiving the basic annual income.^32^ This natural experiment found that implementing an annual income reduced hospitalisation for participants and reduced healthcare utilisation. A different approach to improve the income was carried out in a randomised controlled trial in New Zealand to assess the impact of abolishing prescription co-payment in deprived or low-income groups.^41^ They found that people in the intervention arm had a 30% reduction in the odds of being admitted to hospital during the study year compared with those in the control group. Kim and Shon presented a natural experiment on the impact of ‘medical aid programme’ implementation for low-income households.^47^ They found that those needing medical aid had higher rates of hospitalisation compared with those enrolled in the National Health Insurance. However, the authors hypothesise that this increase in health utilisation is rather a sign of low-income families not having timely access to adequate health services.

### Health and care service interventions

We found five studies reporting the impact of health and care service-based interventions on reducing hospitalisation rates among disadvantaged groups. These interventions were implemented in Australia, Canada, Germany, Spain and Turkey. Two of these studies were rated as strong, and two were rated as weak in our quality assessment.

#### Unemployed persons

One study presented an intervention to reduce hospitalisation for unemployed adults with mental health conditions in Victoria (Australia) through a crisis assessment and treatment service.^35^ The sample group was small (n=69), and the study was not powered to detect a difference in the effect of the interventions contributing to its moderate study quality. However, the study showed a reduction in readmission post-intervention (X^2^, 1.16; p=0.212).

#### Low-income population

Four studies evaluated interventions for specific low-income, urban populations and showed some evidence of impact. All interventions aimed to improve health education and access to planned healthcare using a multidisciplinary approach particularly designed for the target population in question. Lichtl *et al* implemented a community-based intervention comprising walk-in clinics for asylum seekers.^39^ Walk-in health clinics were established throughout reception centres in Germany. The results showed that introducing this service may be effective in reducing avoidable hospitalisations (IRR: 0.80, CI: 0.65 to 1.00; p=0.054), but the effect was attenuated after adjustment analysis for sex, age and admission time. In Canada, Salvalaggio *et al* presented the impact of the Hospital Addiction Medicine Consult Team where a multidisciplinary team with expertise in addiction medicine ensured the continuity of care between hospital and community, ensuring timely access to secondary care as needed as well as supporting the post-discharge journey.^34^ The quasi-experimental assessment, which was rated to have a weak quality, did not find a reduction in hospital admissions after adjusting for covariables (OR: 0.89, 95% CI: 0.55 to 1.45; p=0.633).

We found two interventions that aimed to improve health literacy among groups with low education levels. A quasi-experimental study examining parental discharge training and home visits 3 months after discharge for deprived children with asthma in Turkey showed that health education interventions reduced hospitalisation from 63.3% to 23.4%.^48^ However, this study presented a small sample size, and the analysis was not adjusted for appropriate mediators such as severity of asthma or comorbidities. A similar intervention looking at post-discharge health education consisted of a robust randomised control trial to test the effect of a pharmaceutical care programme delivered post-discharge to patients with low education levels.^46^ The intervention group was re-admitted less than the control group by 54% at 2 months, 42% at 6 months and 32% at 12 months (hazard ratio (HR): 0.56, 95% CI: 0.32 to 0.97).

### Integrative interventions

10 studies implemented integrative interventions across Australia, Canada, France, Germany, Israel, Italy, New Zealand and the United Kingdom. Four of these studies presented interventions that target groups facing housing insecurity, and six studies targeted low-income populations.

#### Housing insecurity

Five studies targeted a population experiencing housing insecurity using an integrative intervention. The evidence was strong or moderate in quality. In France, a randomised controlled trial evaluated a Housing First (HF) intervention among adults experiencing absolute homelessness or precariously housed with a diagnosis of schizophrenia or bipolar disorder.^43^ The findings showed that the HF model combining immediate access to housing and the support of an Assertive Community Treatment team did not impact hospital admissions (RR: 0.96, 95% CI: 0.7 to 1.21).

A similar intervention focused on housing improvements in New Zealand evaluated the ‘Health Housing Programme,’ which focused on (1) improving tenant access to healthcare services, (2) reducing the risk of housing-related health issues by improving the housing environment including insulation and ventilation and (3) identifying social issues and referring to appropriate social service agencies. For people aged 5–34 years, the adjusted HR for acute hospitalisation was 0.77 (95% CI: 0.70 to 0.85) for acute hospitalisations compared with the counterfactual (pre-intervention) data.

A cohort control study explored the effect of a respite facility for adults experiencing primary homelessness, living in tenuous circumstances or marginalised housing on discharge from the hospital in Australia.^36^ This study was judged to be robust and found a reduction in the absolute unplanned inpatient admissions after the introduction of the medical respite centre.

Hwang *et al* presented a similar intervention using supportive housing in a cohort study.^33^ In this study, the intervention was subsidised housing with on-site social care—for adults who were experiencing homelessness or in an emergency shelter or hostel or staying temporarily with others and were financially disadvantaged. The intervention did not reduce the rate of hospitalisations in a pre- and post-analysis (p=0.85).

One study presented an evaluation of a multidisciplinary hospital in-reach programme to reduce admissions among PEH in the United Kingdom.^31^ The intervention involved a risk assessment initially to identify patients’ needs, and this determined the level of intensiveness that the intervention would carry. The intervention ranged from signposting to services such as a housing charity to ongoing support in the community post-discharge for those with the highest level of needs. During the 12-month intervention period, hospital readmissions reduced by approximately 69% compared with the 12-month period prior to Hospital In-reach referral (p<0.01).

#### Low-income population

Five studies evaluated integrated interventions for people living in deprived areas, including for the general population, or those with cardiovascular disease or asthma. The results of these studies showed moderate quality evidence of a decrease in hospitalisations; one weak study showed no evidence of change. Downing *et al* used a longitudinal matched controlled study to test the effect of a ‘one-stop’ consultant-led service integrated with a nurse-led heart failure clinic for patients with cardiovascular disease in a highly deprived region of the United Kingdom. Through matching with regions with similar characteristics that acted as control groups, they found that the integrative community-based service was associated with a lower rate of emergency cardiovascular disease admissions in a deprived population (difference-in-differences: −59.92, 95% CI: −96.19 to −23.64; p=0.001).

During 2015–2016, Horwitz *et al* carried out a coaching programme on asthma control across low socioeconomic groups in Israel, defined as increased resident-to-room ratio, unemployed or living outside the city.^44^ The prospective cohort study of weak quality found no decrease in healthcare utilisation in the intervention group compared with the control (p=0.98).

Castriotta *et al* presented an integrative intervention known as ‘Habitat Microaree’ to facilitate discharge planning and access to social services and outpatient facilities across deprived neighbourhoods in Italy, as per national deprivation index measures.^45^ The intervention assessed priority health problems, provided home healthcare, facilitated collaboration across institutions and fostered a participatory community. The intervention reduced the rate of hospitalisations for all causes in this area compared with non-participants (HR: 0.95, 95% CI: 0.91 to 0.99).

A UK-based study used retrospective cross-over design to evaluate the use of a Multi-Disciplinary Group to deliver an integrated contact centre and support high-risk patients on discharge to community services.^30^ This study, judged to be moderate quality, reported that planned admissions to hospital decreased. A similar intervention, reported in a moderate quality study, evaluated the use of health and social care providers coordinating access to services for high-risk patients in Germany. Ress *et al* found increases in hospital admissions and healthcare utilisation as access improved, likely owing to underlying unmet need.^40^

## Discussion

This systematic review identified 20 studies carried out across OECD countries with public universal health coverage that examined interventions targeting hospitalisation and readmissions in socioeconomically disadvantaged groups. We present interventions across three domains and targeted different socioeconomically disadvantaged groups. The interventions were implemented in countries in European countries as well as Canada, Australia, New Zealand and South Korea.

We found evidence that population health and policy interventions at local, regional and national level can reduce health inequalities in hospitalisations.^29 32 41 49^ Our findings highlight that interventions to improve housing standards, income and access to healthcare all reduced the need for hospitalisations. We hypothesise that these interventions lead to a healthier population by improving the quality of life and reducing poverty, minimising the impact of health inequalities. Certain interventions, such as those targeting housing standards, may also deliver short-term benefits by reducing the exacerbation of pre-existing conditions, for example, reducing exposure to mould and damp that contribute to asthma exacerbations, or by minimising immediate risks, such as installing safety features to prevent falls. These interventions can lead to more immediate reduction in hospital admissions.

Health and care service-based interventions yield mixed results, with many interventions not impacting hospital admissions.^34 35 39^ The health and care service-based interventions reported in the literature were often designed to address one specific aspect of patient’s health, and we believe this may impact on how sustainable and effective these interventions are. At the same time, health and care service-based interventions may uncover unmet health needs and lead to an increase in hospital admissions in the short term. We expect that the overall rate of hospitalisations would decrease once health needs are identified and appropriately addressed. Similar findings have been reported in the literature but not specific to socioeconomically disadvantaged populations, where self-management, empowerment and integration led to a reduction in overall hospitalisation across the whole population in the long term.^50^

Studies reporting on integrative interventions also found mixed results. We hypothesise that this may be related to the follow-up time of the studies. Some interventions that improved access to healthcare services by reducing costs led to an increase in the number of hospital admissions.^40^ Most integrative interventions were evaluated at 18 months to 2 years, and it may have been too early to appreciate their impact. We hypothesise that increases in hospitalisation rates that occur in the short term may be due to unmet healthcare needs present within the population. If such interventions had a long-term evaluation, a reduction in hospitalisations may have been observed.

We urge that governments and relevant stakeholders prioritise interventions to address the wider social, economic and environmental determinants of health to improve health and well-being alongside interventions to improve the equity of access, experience and outcome of healthcare to achieve reductions in hospitalisations. Our findings support addressing areas such as housing, income and access to healthcare to improve population health. However, these improvements may only be evident in long-term follow-up, and this may be why integrative interventions appear to be less effective in our results.

Previous literature found a lack of evidence to support many of the interventions implemented.^51 52^ We hope our findings evidencing that interventions targeting socioeconomically disadvantaged groups can help to reduce hospitalisations will inform future evidence-based policy making. Population health and policy interventions may be used to buffer the wider impacts of social and economic policy, while health and care service-based interventions can improve access, acceptability, availability and quality of health services to reduce hospitalisations and re-hospitalisations. Integrative interventions may bring policy change while also driving improvements in health and care services to reduce socioeconomic inequalities in hospital admissions. Going forward, coordinated approaches that include interventions that span all domains presented in this analysis may be the most effective at reducing hospitalisations among socioeconomically disadvantaged groups.

It was notable that very few studies identified in the search strategy reported on interventions focused on socioeconomically disadvantaged groups. A similar problem has been found in clinical trial reporting.^53^ Similarly, service users, people with lived experiences and members of the public were only reported to be involved in the design of one intervention. Public and patient engagement and coproduction of intervention can be key to obtaining positive results, and future interventions should involve service users across all stages.^54^

### Strengths and limitations

This comprehensive systematic review explored international literature with no restrictions on language to capture as many articles as possible. The search strategy used an equity filter which has been previously validated, adding strength to our work. Similarly, we screened a vast number of records and double-screened full-texts to ensure all literature that met the inclusion criteria was captured. We also sought advice from experts and searched grey literature to strengthen our search. Other strengths of this review include our critical appraisal of the articles using a validated tool and a robust narrative synthesis approach to bring findings together following PRISMA-E and SWiM guidelines.

The three domains used to categorise interventions in this review were agreed through an iterative consultation that involved all authors and the UNFAIR expert advisory panel, which included patient and public representation (http://bit.ly/UNFAIRstudy). In the published protocol, we had originally planned to categorise into four domains but found in reality that all community-based interventions identified were all also integrative interventions, so in consultation with our expert advisory panel, we amalgamated these domains together. This demonstrates how we worked with a flexible theoretical framework, and due to the nature of social and public health interventions, it is not mutually exclusive. Hence, interventions may fit into more than one category, but for the purposes of the analysis, they were classified into one of three domains. Categorisation of the interventions by domain was agreed by authors carrying out data extraction (BNM, JO and WB), and any disagreements were resolved through consultation with a fourth author (SS).

However, several limitations should be considered. First, the search strategy does not include population-specific search terms, and this may mean that studies reporting on interventions for certain socioeconomically marginalised groups may be under-represented through our search strategy. To mitigate this, we used a previously validated equity filter which employs a wide range of equity-focused search terms.^22^ Second, the vast number of hits yielded from our search strategy meant that it was not feasible to double screen all abstracts; however, there was high concordance in screening across the 10% that were double screened. Third, by focusing on hospitalisations as the outcome of interest, we made an implicit assumption that all hospitalisations are potentially avoidable if certain interventions were implemented on time. However, we know that some hospitalisations will be necessary and appropriate to meet patients’ needs. Finally, many interventions that aim to reduce hospitalisations in vulnerable groups may be implemented at a local or grassroots level, and so, these may not be disseminated through academic publications or reports. Given this, our search may be limited and not reflect all interventions that are carried out in this area of work, although we mitigated this by searching grey literature.

When it comes to synthesising our findings, the range of intervention types, definitions of ‘hospitalisation,’ target populations and outcome measures led to heterogeneity of studies which precluded meta-analysis. The lack of an internationally agreed definition for terms such as ‘hospitalisation’ or ‘socioeconomically disadvantaged population’ contributes to the degree of heterogeneity. We used vote counting methodology, which considers neither statistical significance nor the size of the effect and does not account for differences in relative sizes of the studies as an alternative when heterogeneity precluded meta-analyses. So, caution is needed when interpreting the summary direction of effects, as it is difficult to quantify the impact of these interventions, particularly when studies may be using different definitions. This reduces the extent to which firm conclusions can be drawn on the basis of this research alone and points to the need for further research to investigate this important issue. The nature of the populations of interest in our work is very dependent on the wider societal contexts, and so, the findings may not be generalisable to other settings. Recognising these limitations, our study adds to previous evidence demonstrating the importance of addressing wider social determinants such as housing to improve population health, while acknowledging the sparsity and diversity of the existing evidence in this area which hinders making firm conclusions.^55^,^58^

### Future research and conclusions

Our findings suggest that evidence for interventions to reduce hospitalisations in socioeconomically disadvantaged groups remains limited and further research is needed to explore what interventions work best to reduce hospitalisation. It is important that all studies evaluating interventions to reduce hospital admission report their findings by socioeconomic status and report on long-term outcomes, particularly for interventions that may improve access to healthcare and drive an initial increase in healthcare utilisation followed by stabilisation and reduction. New interventions should be designed with active involvement of the target groups and then tested using robust methodology such as a randomised trial or a cohort study that explored pre- and post-differences in admissions. In addition, spaces where best practice examples can be shared would be beneficial to continue to foster cross-sector collaboration to contribute towards the development of multicomponent interventions. We would encourage further research into integrative interventions that address social determinants of health in a holistic way in combination with policy changes that improve purchasing power and empower individuals to live a healthy life as a way to reduce hospital admissions. In the future, particular attention should also be given to the intersectionality that exists between different social determinants of health, for example, PEH and mental health problems or racial/ethnic inequalities, and bespoke interventions should be designed to meet their specific needs.

As economies across the world experience economic uncertainty, populations will face increased social inequality and hospitalisation rates. It is important for national, regional and local initiatives to be designed and implemented to avert any further widening of these health inequalities. We know that, as health services and societies become more strained, disadvantaged groups who may lack social protections or welfare networks suffer the most. In view of this double burden, it is imperative to take a proactive approach to provide timely access to high-quality healthcare as well as appropriate housing, food security, education and employment. To achieve the necessary reduction in hospitalisations for health service sustainability, those who plan, pay for and deliver health services need to work with government as well as policy makers, social care providers and service users to identify and address the social determinants of health.

## Supplementary material

10.1136/bmjph-2025-002592 online supplemental file 1

## Data Availability

No data are available.
