# Supplementary material for: Social and policy interventions to reduce hospital admissions among socioeconomically disadvantaged groups in OECD countries with universal health care: a systematic review
Source: BMJ Public Health. 2025 Sep 23;3(2):e002592. doi: 10.1136/bmjph-2025-002592 (PMC12458780; doi:10.1136/bmjph-2025-002592)
Supplement: online supplemental file 1 [file bmjph-3-2-s001.pdf]

Supplementary Files

Content:

Supplementary File 1: MEDLINE Search Strategy
Supplementary File 2: UNFAIR Project team and collaborators.
Supplementary File 3: PRISMA Checklist
Supplementary File 4: Synthesis Without Meta-analysis (SWiM)
Supplementary File 5: Reasons for study exclusion after full-text review
Supplementary File 6: Summary of studies

Supplementary File 1: MEDLINE Search Strategy

1	Hospitalization/
2	Patient Readmission/
3	Patient Admission/
4	((emergency or unplanned or unanticipated or unexpected or avoid*) adj3 (admission* or readmission* or hospitali#ation*)).ti,ab,kw.
5	(overnight stay adj3 admission*).ti,ab,kw.
6	(primary care adj3 admission*).ti,ab,kw.
7	(ambulatory care adj3 admission*).ti,ab,kw.
8	or/1-7
9	((program* or policy or policies or strateg* or scheme* or intervention* or project* or initiative*) adj5 (evaluat* or effect* or measur* or assess* or experiment* or impact*)).ti,ab,kw.
10	(comparative study or controlled clinical trial or evaluation studies or meta analysis or pragmatic clinical trial or randomized controlled trial or "systematic review").pt.
11	exp Clinical Trial/ or exp Randomized Controlled Trial/ or exp Randomization/ or Random Allocation/ or Double-Blind Method/ or Single-Blind Method/ or exp Cross-Over Studies/ or Program Evaluation/
12	(RCT or randomi* or nonrandomi* or non randomi* or quasiexperiment* or quasi experiment* or quasirandomi* or quasi randomi* or pseudoexperiment* or pseudo experiment* or pseudorandomi* or pseudo randomi* or natural experiment* or pretest or pre test or posttest or post test or time series or repeat* measure* or systematic review*).ti,ab,kw.
13	(before adj1 after adj1 (stud* or trial* or design*)).ti,ab.
14	or/9-13
15	8 and 14
16	(Algeria* or Egypt* or Liby* or Morocc* or Tunisia* or Western Sahara* or Angola* or Benin or Botswana* or Burkina Faso or Burundi or Cameroon or Cape Verde or Central African Republic or Chad or Comoros or Congo or Djibouti or Eritrea or Ethiopia* or Gabon or Gambia* or Ghana or Guinea or Keny* or Lesotho or Liberia or Madagasca* or Malawi or Mali or Mauritania or Mauritius or Mayotte or Mozambiq* or Namibia* or Niger or Nigeria* or Reunion or Rwand* or Saint Helena or Senegal or Seychelles or Sierra Leone or Somalia or South Africa* or Sudan or Swaziland or Tanzania or Togo or Ugand* or Zambia* or Zimbabw* or China or Chinese or Hong Kong or Macao or Mongolia* or Taiwan* or Belarus or Moldov* or Russia* or Ukraine or Afghanistan or Armenia* or Azerbaijan or Bahrain or Cyprus or Cypriot or Georgia* or Iran* or Iraq* or Israel* or Jordan* or Kazakhstan or Kuwait or Kyrgyzstan or Leban* or Oman or Pakistan* or Palestin* or Qatar or Saudi Arabia or Syria* or Tajikistan or Turkmenistan or United Arab Emirates or Uzbekistan or Yemen or Bangladesh* or Bhutan or British Indian Ocean Territory or Brunei Darussalam or Cambodia* or India* or Indonesia* or Lao or People's Democratic Republic or Malaysia* or Maldives or Myanmar or Nepal or Philippin* or Singapore or Sri Lanka or Thai* or Timor Leste or Vietnam or Albania* or Andorra or Bosnia* or Herzegovina* or Bulgaria* or Croatia* or Estonia or Faroe Islands or Greenland or Liechtenstein or Lithuani* or Macedonia or Malta or maltese or Romania or Serbia* or Montenegro or Slovenia or Svalbard or Argentina* or Belize or Bolivia* or Brazil* or chile or Chilean or Colombia* or Costa Rica* or Cuba or Ecuador or El Salvador or French Guiana or Guatemala* or Guyana or Haiti or Honduras or Jamaica* or Nicaragua* or Panama or Paraguay or Peru or Puerto Rico or Suriname or Uruguay or Venezuela or developing countr* or south America*).ti,sh.
17	15 not 16
18	limit 17 to (english language and humans and yr="1999 -Current")
19	Residence Characteristics/
20	Environment design/
21	exp Marital status/
22	neighbo?rhood*.mp.
23	residential environment*.mp.
24	rural*.mp.
25	inner?city.mp.
26	housing instability.mp.

27	housing insecurity.mp.
28	housing strain.mp.
29	housing security.mp.
30	mortgage problems.mp.
31	foreclosure.mp.
32	eviction*.mp.
33	housing loss.mp.
34	home repossession*.mp.
35	home ownership.mp.
36	(repossess* adj3 hous*).mp.
37	(repossess* adj3 propert*).mp.
38	mortgage delinquency.mp.
39	mortgage arrears.mp.
40	mortgage debt*.mp.
41	overcrowding.mp.
42	(living adj1 (outside or inside or near* or adjacent)).mp.
43	(household adj2 size).mp.
44	(marital status or marriage status).mp.
45	(widow* or cohabit* or divorce* or single parent* or live* alone).mp.
46	or/19-45
47	Occupations/
48	Unemployment/
49	occupations.mp.
50	unemployment.mp.
51	or/47-50
52	exp Educational status/
53	Education/
54	Schooling.mp.
55	educational status.mp.
56	(education* adj2 level?).mp.
57	((higher or better or worse or less) adj educated).mp.
58	((higher or better or worse or less) adj level? of education).mp.
59	or/52-58
60	Social determinants of Health/
61	Psychosocial Deprivation/
62	Sociological Factors/
63	Working Poor/
64	Hierarchy, Social/
65	disparit*.mp.
66	inequalit*.mp.
67	inequit*.mp.
68	equity.mp.
69	deprivation.mp.
70	gini.mp.
71	concentration index.mp.
72	Socioeconomic Factors/
73	Social Welfare/
74	exp Social Class/
75	exp Poverty/
76	Income/
77	Social class*.mp.

78	social determinants.mp.
79	social status.mp.
80	social position.mp.
81	social background.mp.
82	social circumstance*.mp.
83	socio-economic.mp.
84	socioeconomic.mp.
85	sociodemographic.mp.
86	socio-demographic.mp.
87	SES.mp.
88	disadvantaged.mp.
89	impoverished.mp.
90	poverty.mp.
91	economic level.mp.
92	assets index.mp.
93	income*.mp.
94	or/60-93
95	Social Stigma/
96	social capital/
97	Social Control, Informal/
98	exp Social Support/
99	exp Social Environment/
100	Trust/
101	Social conditions/
102	Social isolation/
103	Social marginalization/
104	Anomie/
105	social participation/
106	social exclusion.mp.
107	(social adj (capital or cohes* or organis* or organiz*)).mp.
108	(community adj3 (cohes* or participa*)).mp.
109	((neighbourhood or neighborhood) adj cohes*).mp.
110	social relationships.mp.
111	social network*.mp.
112	collective efficacy.mp.
113	civil society.mp.
114	informal social control.mp.
115	neighbo*rhood disorder.mp.
116	social disorgani?ation.mp.
117	anomie.mp.
118	social support.mp.
119	social participation.mp.
120	trust.mp.
121	emotional support.mp.
122	psychosocial support.mp.
123	community capital.mp.
124	neighbo*rhood cohesion.mp.
125	social influence.mp.
126	(soci*context* or soci*-context*).mp.
127	or/95-126
128	Health Status Disparities/

129	Health Services Accessibility/
130	Health Equity/
131	health*care disparit*.mp.
132	health care disparit*.mp.
133	health status disparit*.mp.
134	health disparit*.mp.
135	health inequalit*.mp.
136	health inequit*.mp.
137	medically underserved.mp.
138	or/128-137
139	potential determinants.mp.
140	significant correlates of.mp.
141	(independent correlates or independent association*).mp.
142	variables associated with.mp.
143	determinants of.mp.
144	factors associated with.mp.
145	identif* determinants.mp.
146	(more likely or less likely or just as likely).mp.
147	risk factors for.mp.
148	(significantly related to or significant predictor).mp.
149	(also adj2 associated with).mp.
150	(at increased risk or at decreased risk).mp.
151	association* between.mp.
152	(positively associated or negatively associated).mp.
153	differed by.mp.
154	(were high* amongst or were low* amongst).mp.
155	(inverse relationship with or inversely associated with or inversely related to).mp.
156	reverse association.mp.
157	differentially affects.mp.
158	evidence of a link between.mp.
159	(significantly adj3 likelihood of).mp.
160	protective factors for.mp.
161	(differ* adj2 according to).mp.
162	(inverse adj2 gradient).mp.
163	(positive adj2 gradient).mp.
164	(negative adj2 gradient).mp.
165	(trends were adj3 across).mp.
166	(related to adj3 variable*).mp.
167	(differences were adj3 explained by).mp.
168	(significant among or no# significant among).mp.
169	or/139-168
170	46 or 51 or 59 or 94 or 127 or 138 or 169
171	18 and 170

Supplementary 2: UNFAIR Project team and collaborators. Updated list available at https://sites.google.com/nihr.ac.uk/unfairstudy/who-is-involved/collaborators?authuser=0

Supplementary 3: PRISMA Checklist

Section and Topic	Item #	Checklist item	Location where item is reported
**TITLE**			
Title	1	Identify the report as a systematic review.	P1
**ABSTRACT**			
Abstract	2	See the PRISMA 2020 for Abstracts checklist.	P2
**INTRODUCTION**			
Rationale	3	Describe the rationale for the review in the context of existing knowledge.	P3
Objectives	4	Provide an explicit statement of the objective(s) or question(s) the review addresses.	P3
**METHODS**			
Eligibility criteria	5	Specify the inclusion and exclusion criteria for the review and how studies were grouped for the syntheses.	P3-4
Information sources	6	Specify all databases, registers, websites, organisations, reference lists and other sources searched or consulted to identify studies. Specify the date when each source was last searched or consulted.	P3-4
Search strategy	7	Present the full search strategies for all databases, registers and websites, including any filters and limits used.	P3-4
Selection process	8	Specify the methods used to decide whether a study met the inclusion criteria of the review, including how many reviewers screened each record and each report retrieved, whether they worked independently, and if applicable, details of automation tools used in the process.	P3-4
Data collection process	9	Specify the methods used to collect data from reports, including how many reviewers collected data from each report, whether they worked independently, any processes for obtaining or confirming data from study investigators, and if applicable, details of automation tools used in the process.	P3-4
Data items	10a	List and define all outcomes for which data were sought. Specify whether all results that were compatible with each outcome domain in each study were sought (e.g. for all measures, time points, analyses), and if not, the methods used to decide which results to collect.	P3-4
	10b	List and define all other variables for which data were sought (e.g. participant and intervention characteristics, funding sources). Describe any assumptions made about any missing or unclear information.	P3-4 Supplement
Study risk of bias assessment	11	Specify the methods used to assess risk of bias in the included studies, including details of the tool(s) used, how many reviewers assessed each study and whether they worked independently, and if applicable, details of automation tools used in the process.	P4-5
Effect measures	12	Specify for each outcome the effect measure(s) (e.g. risk ratio, mean difference) used in the synthesis or presentation of results.	P4-5
Synthesis methods	13a	Describe the processes used to decide which studies were eligible for each synthesis (e.g. tabulating the study intervention characteristics and comparing against the planned groups for each synthesis (item #5)).	P4-5

Section and Topic	Item #	Checklist item	Location where item is reported
	13b	Describe any methods required to prepare the data for presentation or synthesis, such as handling of missing summary statistics, or data conversions.	P4-5
	13c	Describe any methods used to tabulate or visually display results of individual studies and syntheses.	P4-5
	13d	Describe any methods used to synthesize results and provide a rationale for the choice(s). If meta-analysis was performed, describe the model(s), method(s) to identify the presence and extent of statistical heterogeneity, and software package(s) used.	P4-5
	13e	Describe any methods used to explore possible causes of heterogeneity among study results (e.g. subgroup analysis, meta-regression).	P4-5
	13f	Describe any sensitivity analyses conducted to assess robustness of the synthesized results.	N/A
Reporting bias assessment	14	Describe any methods used to assess risk of bias due to missing results in a synthesis (arising from reporting biases).	P5
Certainty assessment	15	Describe any methods used to assess certainty (or confidence) in the body of evidence for an outcome.	P5
**RESULTS**			
Study selection	16a	Describe the results of the search and selection process, from the number of records identified in the search to the number of studies included in the review, ideally using a flow diagram.	P6
	16b	Cite studies that might appear to meet the inclusion criteria, but which were excluded, and explain why they were excluded.	P6
Study characteristics	17	Cite each included study and present its characteristics.	P6
Risk of bias in studies	18	Present assessments of risk of bias for each included study.	P22
Results of individual studies	19	For all outcomes, present, for each study: (a) summary statistics for each group (where appropriate) and (b) an effect estimate and its precision (e.g. confidence/credible interval), ideally using structured tables or plots.	P18-22
Results of syntheses	20a	For each synthesis, briefly summarise the characteristics and risk of bias among contributing studies.	P18-22
	20b	Present results of all statistical syntheses conducted. If meta-analysis was done, present for each the summary estimate and its precision (e.g. confidence/credible interval) and measures of statistical heterogeneity. If comparing groups, describe the direction of the effect.	P18-22
	20c	Present results of all investigations of possible causes of heterogeneity among study results.	N/A
	20d	Present results of all sensitivity analyses conducted to assess the robustness of the synthesized results.	N/A
Reporting biases	21	Present assessments of risk of bias due to missing results (arising from reporting biases) for each synthesis assessed.	P18-22
Certainty of	22	Present assessments of certainty (or confidence) in the body of evidence for each outcome assessed.	P18-22

Section and Topic	Item #	Checklist item	Location where item is reported
evidence			
**DISCUSSION**			
Discussion	23a	Provide a general interpretation of the results in the context of other evidence.	P10
	23b	Discuss any limitations of the evidence included in the review.	P10-11
	23c	Discuss any limitations of the review processes used.	P10-11
	23d	Discuss implications of the results for practice, policy, and future research.	P10-12
**OTHER INFORMATION**			
Registration and protocol	24a	Provide registration information for the review, including register name and registration number, or state that the review was not registered.	P2
	24b	Indicate where the review protocol can be accessed, or state that a protocol was not prepared.	P2
	24c	Describe and explain any amendments to information provided at registration or in the protocol.	P2-5
Support	25	Describe sources of financial or non-financial support for the review, and the role of the funders or sponsors in the review.	P1
Competing interests	26	Declare any competing interests of review authors.	P1
Availability of data, code and other materials	27	Report which of the following are publicly available and where they can be found: template data collection forms; data extracted from included studies; data used for all analyses; analytic code; any other materials used in the review.	P1

From: Page MJ, McKenzie JE, Bossuyt PM, Boutron I, Hoffmann TC, Mulrow CD, et al. The PRISMA 2020 statement: an updated guideline for reporting systematic reviews. BMJ 2021;372:n71. doi: 10.1136/bmj.n71

For more information, visit: http://www.prisma-statement.org/

Supplementary 4: Synthesis Without Meta-analysis (SWiM)

The citation for the Synthesis Without Meta-analysis explanation and elaboration article is: Campbell M, McKenzie JE, Sowden A, Katikireddi SV, Brennan SE, Ellis S, Hartmann-Boyce J, Ryan R, Shepperd S, Thomas J, Welch V, Thomson H. Synthesis without meta-analysis (SWiM) in systematic reviews: reporting guideline BMJ 2020;368:l6890 http://dx.doi.org/10.1136/bmj.l6890

**SWiM is intended to complement and be used as an extension to PRISMA**			
**SWiM reporting item**	**Item description**	**Page in manuscript where item is reported**	**Other***
Methods			
**1** Grouping studies for synthesis	1a) Provide a description of, and rationale for, the groups used in the synthesis (e.g., groupings of populations, interventions, outcomes, study design)	P3-4	
	1b) Detail and provide rationale for any changes made subsequent to the protocol in the groups used in the synthesis	P3-4	
**2** Describe the standardised metric and transformation methods used	Describe the standardised metric for each outcome. Explain why the metric(s) was chosen, and describe any methods used to transform the intervention effects, as reported in the study, to the standardised metric, citing any methodological guidance consulted	P3-4	
**3** Describe the synthesis methods	Describe and justify the methods used to synthesise the effects for each outcome when it was not possible to undertake a meta-analysis of effect estimates	P3-4	
**4** Criteria used to prioritise results for summary and synthesis	Where applicable, provide the criteria used, with supporting justification, to select the particular studies, or a particular study, for the main synthesis or to draw conclusions from the synthesis (e.g., based on study design, risk of bias assessments, directness in relation to the review question)	P3-4	
**5** Investigation of heterogeneity in reported effects	State the method(s) used to examine heterogeneity in reported effects when it was not possible to undertake a meta-analysis of effect estimates and its extensions to investigate heterogeneity	N/A	

**6** Certainty of evidence	Describe the methods used to assess certainty of the synthesis findings	P3-4	
**7** Data presentation methods	Describe the graphical and tabular methods used to present the effects (e.g., tables, forest plots, harvest plots). Specify key study characteristics (e.g., study design, risk of bias) used to order the studies, in the text and any tables or graphs, clearly referencing the studies included	P3-4	
Results			
**8** Reporting results	For each comparison and outcome, provide a description of the synthesised findings, and the certainty of the findings. Describe the result in language that is consistent with the question the synthesis addresses, and indicate which studies contribute to the synthesis	P5-6	
Discussion			
**9** Limitations of the synthesis	Report the limitations of the synthesis methods used and/or the groupings used in the synthesis, and how these affect the conclusions that can be drawn in relation to the original review question	P11	

PRISMA=Preferred Reporting Items for Systematic Reviews and Meta-Analyses.
*If the information is not provided in the systematic review, give details of where this information is available (e.g., protocol, other published papers (provide citation details), or website (provide the URL)).

Supplementary 5: Reason for study exclusion after full-text review

Exclude on study type, n=155

Crawford (2021)	A systematic review examining the clinical and health-care outcomes for congenital heart disease patients using home monitoring programmes	2021
Adams (2014)	Implementation of the Re-Engineered Discharge (RED) toolkit to decrease all-cause readmission rates at a rural community hospital.	2014
Agarwal (2019)	Design and rationale for a pragmatic cluster randomized trial of the Cardiovascular Health Awareness Program (CHAP) for social housing residents in Ontario and Quebec, Canada	2019
Ajmera (2015)	Real-world observational study of association between statin medications and COPD-specific outcomes	2015
Al-Khazaali (2016)	Effective Strategies in Reducing Rehospitalizations in Patients With Heart Failure.	2016
Al-Lami (2019)	Testosterone Replacement Therapy and Rehospitalization in Older Men With Testosterone Deficiency in a Postacute Care Setting.	2019
Al-Shehhi (2017)	Rate and predictors of 1-year readmission in tertiary psychiatric hospitals	2017
Albritton (2018)	The Effect Of The Hospital Readmissions Reduction Program On Readmission And Observation Stay Rates For Heart Failure.	2018
Alcain (2019)	THE EFFECT OF A NURSE-DRIVEN PROGRAM UTILIZING IMPLANTABLE PULMONARY ARTERY PRESSURE MONITORING TO REDUCE HOSPITALIZATIONS IN LOW-SOCIOECONOMIC URBAN PATIENTS WITH HEART FAILURE	2019
Alende-Castro (2018)	Q fever in Spain: Description of a new series, and systematic review.	2018
Altice (2020)	Early Cardiac Rehab to Reduce Heart Failure Readmissions.	2020
Ancona (2004)	Differences in access to coronary care unit among patients with acute myocardial infarction in Rome: old, ill, and poor people hold the burden of inefficiency.	2004
Angelelli (2002)	Access to postacute nursing home care before and after the BBA. Balanced Budget Act.	2002
Anonymous (2019)	The Effect of a Nurse-Driven Program Utilizing Implantable Pulmonary Artery Pressure Monitoring to Reduce Hospitalizations in Low-Socioeconomic Urban Patients with Heart Failure	2019
Apter (2017)	A patient advocate to facilitate access and improve communication, care, and outcomes in adults with moderate or severe asthma: Rationale, design, and methods of a randomized controlled trial	2017
Apter (2022)	Clinic navigation and home visits to improve asthma care in low income adults with poorly controlled asthma: Before and during the pandemic.	2022
Aubry (2015)	Housing First for People With Severe Mental Illness Who Are Homeless: A Review of the Research and Findings From the At Home-Chez soi Demonstration Project	2015
Auger (2015)	Pediatric Asthma Readmission: Asthma Knowledge Is Not Enough?	2015
Beckman (2019)	Medicare Annual Wellness Visit Association With Healthcare Quality and Costs.	2019
Bekelis (2017)	Comparison of clipping and coiling in elderly patients with unruptured cerebral aneurysms.	2017
Bell (2014)	Associations of PM2.5 constituents and sources with hospital admissions: analysis of four counties in Connecticut and Massachusetts (USA) for persons >= 65 years of age.	2014
Bell (2015)	Effect of a pharmacist counseling intervention on healthcare utilization after hospital discharge: A randomized controlled trial	2015
Benenson (2017)	Factors influencing utilization of hospital services by adult sickle cell disease patients: a systematic review.	2017
Berger (2020)	Ambulatory Care–Sensitive Conditions Associated With Potentially Avoidable Hospital Admissions.	2020
Berkman (2011)	Health literacy interventions and outcomes: an updated systematic review.	2011
Borza (2017)	Association between hospital accountable care organization status and readmission following cystectomy and other major surgery	2017
Bracken (2003)	The impact of prenatal and postnatal home visiting on utilization of care.	2003
Callahan (2012)	Transitions in Care for Older Adults with and without Dementia	2012
Chambers (2016)	Reducing 30-day Readmission After Joint Replacement.	2016
Chan (2021)	Community-based interventions for childhood asthma using comprehensive approaches: a systematic review and meta-analysis	2021
Chen (2021)	Reducing asthma hospitalisations in at-risk children: A systematic review	2021
Chopra (2017)	Non-adherence to statins and antihypertensive medications and hospitalizations among elderly fee-for-service medicare beneficiaries with pre-existing coronary artery disease and incident cancer	2017

Conway (2006)	Maternal health: does prenatal care make a difference?	2006
Crowley (2020)	Intervention protocol: OPtimising thERapy to prevent avoidable hospital Admission in the Multi-morbid elderly (OPERAM): a structured medication review with support of a computerised decision support system	2020
Cusimano (2012)	Socioeconomic status and hospital admission of team sport-related brain injuries in Canadian youth	2012
Davis (2016)	Coordinating complex care-a high-risk residency clinic	2016
DeWalt (2009)	Comparison of a one-time educational intervention to a teach-to-goal educational intervention for self-management of heart failure: design of a randomized controlled trial	2009
Estrada (2017)	Drug-safety program impact in hospitalization persistent severe asthma patients	2017
Fann (2011)	Maximizing Health Benefits and Minimizing Inequality: Incorporating Local-Scale Data in the Design and Evaluation of Air Quality Policies	2011
Fick (2002)	Delirium superimposed on dementia: a systematic review.	2002
Field (2015)	Association of early post-discharge follow-up by a primary care physician and 30-day rehospitalization among older adults	2015
Fleishmann (1995)	Longitudinal patterns of medical service use and costs among people with AIDS	1995
Fleming (2017)	Educational and Health Outcomes of Children Treated for Attention-Deficit/Hyperactivity Disorder.	2017
Fleming (2019)	Educational and health outcomes of children treated for asthma: Scotland-wide record linkage study of 683716 children	2019
Flinn (2013)	Medical home for persons with disabilities: A target for the triple aim	2013
Folger (2017)	Evaluation of Early Childhood Home Visiting to Prevent Medically Attended Unintentional Injury	2017
Follath (2006)	Beta-blockade today: the gap between evidence and practice	2006
Fonarow (2004)	Organized program to initiate lifesaving treatment in hospitalized patients with heart failure (OPTIMIZE-HF): Rationale and design	2004
Fond (2013)	Self-reported major depressive symptoms at baseline impact abstinence prognosis in smoking cessation program. A one-year prospective study	2013
Foraker (2008)	Neighborhood income, health insurance, and prehospital delay for myocardial infarction: the atherosclerosis risk in communities study.	2008
Ford (2015)	Top ten risk factors for morbidity and mortality in patients with chronic systolic heart failure and elevated heart rate: The SHIFT Risk Model.	2015
Foster (2018)	Molecular Characterization of Staphylococcus aureus Isolates From Children With Periorbital or Orbital Cellulitis.	2018
Fox (2013)	Increased perioperative b-type natriuretic peptide associates with heart failure hospitalization or heart failure death after coronary artery bypass graft surgery	2013
France (2001)	Smoking cessation interventions among hospitalized patients: what have we learned?.	2001
Francis (2004)	Acute heart failure: patient management of a growing epidemic.	2004
Franklin (2020)	A modelling-based economic evaluation of primary-care-based fall-risk screening followed by fall-prevention intervention: a cohort-based Markov model stratified by older age groups.	2020
Freedman (2013)	Treatment of acute gastroenteritis in children: an overview of systematic reviews of interventions commonly used in developed countries.	2013
Freeman (2012)	Do sales of pseudoephedrine predict methamphetamine-related hospitalizations?	2012
French (2006)	National Veterans Health Administration hospitalizations for syncope compared to acute myocardial infarction, fracture, or pneumonia in community-dwelling elders: outpatient medication and comorbidity profiles.	2006
Frencher (2010)	A comparative analysis of serious injury and illness among homeless and housed low income residents of New York City.	2010
Friedman (2018)	Association Between Left Atrial Appendage Occlusion and Readmission for Thromboembolism Among Patients With Atrial Fibrillation Undergoing Concomitant Cardiac Surgery.	2018
Froman (2005)	Randomized study of stability and change in patients' advance directives.	2005
Fu (2018)	Mean medical costs associated with vaginal and vulvar cancers for commercially insured patients in the United States and Texas.	2018
Furre (2014)	Characteristics of adolescents subjected to restraint in acute psychiatric units in Norway: a case-control study.	2014
Gaalema (2019)	The effect of executive function on adherence with a cardiac secondary prevention program and its interaction with an incentive-based intervention	2019
Galan (2015)	Assessing the effects of the Spanish partial smoking ban on cardiovascular and respiratory diseases: methodological issues.	2015
Galbraith (2011)	Cost Analysis of a Falls-prevention Program in an Orthopaedic Setting	2011
Galbraith (2017)	Long-Term Impact of a Postdischarge Community Health Worker Intervention on Health Care Costs in a Safety-Net System.	2017

Gali (2019)	Anemia profile in heart failure: High prevalence of iron deficiencyand scope for correction. a performance improvement project	2019
Gallagher (2011)	Social support and self-care in heart failure.	2011
Galletly (2011)	Bed accessibility in a private psychiatric hospital.	2011
Galloway (2016)	Hospital Readmission Following Discharge From Inpatient Rehabilitation for Older Adults With Debility.	2016
Galvin (2017)	Adverse outcomes in older adults attending emergency departments: a systematic review and meta-analysis of the Identification of Seniors At Risk (ISAR) screening tool.	2017
Galyean (2009)	Previous cesarean section and the risk of postpartum maternal complications and adverse neonatal outcomes in future pregnancies.	2009
Gambassi (2000)	Management of heart failure among very old persons living in long-term care: Has the voice of trials spread?	2000
Gamble (2010)	Admission hypoglycemia and increased mortality in patients hospitalized with pneumonia.	2010
Gan (2006)	The timed up and go test does not predict length of stay on an acute geriatric ward	2006
Ganapathi (2014)	Frailty and risk in proximal aortic surgery.	2014
Gandhi (2014)	Emergency department visit classification using the NYU algorithm.	2014
Gant (2019)	Retrospective evaluation of factors influencing transfusion requirements and outcome in cats with pelvic injury (2009-2014): 122 cases	2019
Gao (2011)	Evaluation of Dronedarone Use in the US Patient Population Between 2009 and 2010: A Descriptive Study Using a Claims Database	2011
Gao (2016)	Multifaceted interventions reduce 30-day hospital readmissions in at-risk medicaid members from four states and the district of Columbia	2016
Gao (2020)	Predictive Value of the Acute-to-Chronic Glycemic Ratio for In-Hospital Outcomes in Patients With ST-Segment Elevation Myocardial Infarction Undergoing Percutaneous Coronary Intervention.	2020
Gardiner (2019)	Acute circulatory complications in people with diabetes mellitus type 2: How admission varies between urban and rural Victoria.	2019
Gardner (2002)	Improvement in the undertreatment of osteoporosis following hip fracture	2002
Gardner (2009)	Clinical signs of infection in diabetic foot ulcers with high microbial load	2009
Gaskin (2000)	Racial and ethnic differences in preventable hospitalizations across 10 states.	2000
Gates (2021)	Health inequities related to vaccination: An evidence map of potentially influential factors and systematic review of interventions	2021
Gay (2019)	Association of Extending Hospital Length of Stay With Reduced Pediatric Hospital Readmissions.	2019
Geelhoed (2012)	Emergency department overcrowding, mortality and the 4-hour rule in Western Australia.	2012
Gerald (2010)	Cost-effectiveness of school-based asthma screening in an urban setting	2010
Gerding (2018)	Bezlotoxumab for Prevention of Recurrent Clostridium difficile Infection in Patients at Increased Risk for Recurrence.	2018
Gerhardsson (2015)	Asthma and Chronic Obstructive Pulmonary Disease Overlap Syndrome: Doubled Costs Compared with Patients with Asthma Alone.	2015
Gerke (2014)	Association of hospitalizations for asthma with seasonal and pandemic influenza.	2014
Ghaderi (2019)	Hospitalization following influenza infection and pandemic vaccination in multiple sclerosis patients: a nationwide population-based registry study from Norway	2019
Ghanayem (2012)	Interstage mortality after the Norwood procedure: Results of the multicenter Single Ventricle Reconstruction trial	2012
Gheorghiade (2005)	Rationale and design of the multicenter, randomized, double-blind, placebo-controlled study to evaluate the Efficacy of Vasopressin antagonism in Heart Failure: outcome Study with Tolvaptan (EVEREST)	2005
Gianfrancesco (2007)	Hospitalization risks in the treatment of bipolar disorder: comparison of antipsychotic medications.	2007
Gibson (2013)	A systematic review of evidence on the association between hospitalisation for chronic disease related ambulatory care sensitive conditions and primary health care resourcing.	2013
Giday (2019)	Factors other than medical acuity that influence hospitalisation: A scoping review protocol	2019
Gidwani (2015)	Association Between Acute Medical Exacerbations and Consuming or Producing Web-Based Health Information: Analysis From Pew Survey Data.	2015
Gijsberts (2017)	Effect of Monocyte-to-Lymphocyte Ratio on Heart Failure Characteristics and Hospitalizations in a Coronary Angiography Cohort.	2017
Gill (2006)	Bathing disability and the risk of long-term admission to a nursing home.	2006
Gillard (2015)	Denial of risk: The effects of positive impression management on risk assessments for psychopathic and nonpsychopathic offenders	2015

Gillis (1997)	Factors associated with unplanned discharge from psychiatric day treatment programs - A multicenter study	1997
Gilotra (2015)	Reasons for heart failure hospitalization: Nonadherence versus worsening heart failure	2015
Gilstrap (2019)	Association Between Clinical Practice Group Adherence to Quality Measures and Adverse Outcomes Among Adult Patients With Diabetes.	2019
Givertz (2014)	Renal Function Trajectories and Clinical Outcomes in Acute Heart Failure	2014
Glasziou (2002)	Cholesterol-lowering therapy with pravastatin in patients with average cholesterol levels and established ischaemic heart disease: is it cost-effective?.	2002
Gleason (2017)	Effect of drinking water source on associations between gastrointestinal illness and heavy rainfall in New Jersey.	2017
Gleason (2020)	Health navigators are an effective strategy to bridge the gap for school-aged children at risk for asthma disparities	2020
Go (2006)	Hemoglobin level, chronic kidney disease, and the risks of death and hospitalization in adults with chronic heart failure - The anemia in chronic heart failure: Outcomes and Resource Utilization (ANCHOR) Study	2006
Goeree (2013)	Economic appraisal of a community-wide cardiovascular health awareness program	2013
Goff (2014)	Project Buena Salud: Evaluation of an enhanced primary care program for low-income hispanic patients with type 2 diabetes	2014
Goforth (2015)	Exertional Heat Stroke in Navy and Marine Personnel: A Hot Topic	2015
González (2022)	Colorectal Cancer Screening in Castilla La Mancha, Spain: The Influence of Social, Economic, Demographic and Geographic Factors.	2022
Greene (2018)	Outpatient use of intravenous diuretics for treatment of heart failure: Temporal trends and associations with 30-day mortality and readmission following hospitalization for heart failure	2018
Griffin (2000)	Nonsteroidal antiinflammatory drugs and acute renal failure in elderly persons	2000
Hilts (2021)	Hospital Partnerships for Population Health: A Systematic Review of the Literature	2021
Hungerford (2018)	Influenza-associated hospitalisation, vaccine uptake and socioeconomic deprivation in an English city region: an ecological study	2018
Ikram-Bashir (2012)	Designing a behavioural-educational intervention using intervention mapping to reduce the high rates of paediatric asthma hospital admissions in an inner-city area of birmingham	2012
Jiang (2021)	Impact of High Deductible Health Plans on Diabetes Care Quality and Outcomes: Systematic Review	2021
Kaneko (2019)	Associations of Patient Experience in Primary Care With Hospitalizations and Emergency Department Visits on Isolated Islands: A Prospective Cohort Study.	2019
Kessing (2011)	Investigating a TELEmedicine solution to improve MEDication adherence in chronic Heart Failure (TELEMED-HF): study protocol for a randomized controlled trial	2011
Kiil (2014)	How does copayment for health care services affect demand, health and redistribution? A systematic review of the empirical evidence from 1990 to 2011.	2014
LeBreton (2015)	Implementation of a validated health literacy tool with teach-back education in a super utilizer patient population.	2015
Li (2017)	An APN-led transitional care program to reduce 30-day readmissions in patients with heart failure	2017
Madden (2022)	Early implementation of the structured medication review in England: a qualitative study	2022
McWilliams (2007)	Use of health services by previously uninsured Medicare beneficiaries.	2007
Medicaid Utilization... (Cantor)	Medicaid Utilization and Spending among Homeless Adults in New Jersey: Implications for Medicaid-Funded Tenancy Support Services	
Metcalfe (2018)	Impact of public release of performance data on the behaviour of healthcare consumers and providers	2018
Miaozhen (2017)	Using an APN-Led Transitional Care Program to Reduce 30-Day Hospital Readmissions.	2017
Moledina (2021)	A comprehensive review of prioritised interventions to improve the health and wellbeing of persons with lived experience of homelessness	2021
Nelson (2016)	Peer support for achieving independence in diabetes (peeraid): Results of a randomized controlled trial of community health worker assisted self-management support among low-income adults with diabetes	2016
Obiagwu (2018)	Developing a targeted approach to 30-day chf readmissions	2018
Pare (2007)	Systematic review of home telemonitoring for chronic diseases: the evidence base.	2007
Permanent Supportive... (Yinan)	Permanent Supportive Housing With Housing First to Reduce Homelessness and Promote Health Among Homeless Populations With Disability: A Community Guide Systematic Review.	
Reategui-Sokolova (2018)	A comprehensive care program reduces the number and length of hospitalizations in systemic lupus erythematosus patients	2018
Rice (2023)	Development and Implementation of a Maryland State Program Providing Hospital Payment Incentives for Reduction in Readmission Disparities.	2023

Riggs (2009)	The influence of home care nursing visit pattern on heart failure patient outcomes.	2009
Rogstad (2022)	Social Risk Adjustment In The Hospital Readmissions Reduction Program: A Systematic Review And Implications For Policy.	2022
Rust (2013)	Inhaled corticosteroid adherence and emergency department utilization among Medicaid-enrolled children with asthma	2013
Salkever (1999)	Assertive community treatment for people with severe mental illness: The effect on hospital use and costs	1999
Smith (2023)	Racialized economic segregation and potentially preventable hospitalizations among Medicaid/CHIP-enrolled children.	2023
Social Risk Interventions... (Tyris)	Social Risk Interventions and Health Care Utilization for Pediatric Asthma A Systematic Review and Meta-analysis	
Soones (2016)	The mobile acute care team: Preliminary outcomes of a bundled-payment hospital at home program	2016
Stockings (2018)	Whole-of-community interventions to reduce population-level harms arising from alcohol and other drug use: a systematic review and meta-analysis.	2018
Tabit (2017)	A bundled intervention including early consultation with a cardiologist in the emergency department to reduce re-hospitalizations and healthcare cost for high-risk urban patients with acute decompensated heart failure	2017
Telfar (2020)	Renting Poorer Housing: Ecological Relationships Between Tenure, Dwelling Condition, and Income and Housing-Sensitive Hospitalizations in a Developed Country.	2020
The Asthma Linking Project:... (O'Neill)	The Asthma Linking Project: community-based education and support for asthma in a culturally and linguistically diverse population.	
Thomas (2014)	Pharmacist-led interventions to reduce unplanned admissions for older people: a systematic review and meta-analysis of randomised controlled trials.	2014
Trachtenberg (2014)	Inequities in ambulatory care and the relationship between socioeconomic status and respiratory hospitalizations: a population-based study of a canadian city.	2014
Wang (2022)	Study on the Application of the Concept of Childlike Interest with Refined Nursing Intervention in the Treatment of Children with Severe Pneumonia.	2022
Xu (2023)	An Unsupervised Machine Learning Approach to Evaluating the Association of Symptom Clusters With Adverse Outcomes Among Older Adults With Advanced Cancer: A Secondary Analysis of a Randomized Clinical Trial.	2023
Youn (2022)	Disparities in diabetes-related avoidable hospitalization among diabetes patients with disability using a nationwide cohort study.	2022

Exclude on outcome, n=231

Balamurugan (2006)	Barriers to diabetes self-management education programs in underserved rural Arkansas: implications for program evaluation.	2006
Bauer (2021)	Partnering with parents to remove barriers and improve influenza immunization rates for young children	2021
Berge (2022)	Gender specific early treatment for women with alcohol addiction (EWA): Impact on work related outcomes. A 25-year registry follow-up of a randomized controlled trial (RCT).	2022
Bhaumik (2019)	Community asthma initiative: Cost analyses using claims data from a Medicaid managed care organization	2019
Bindman (2001)	Primary and secondary care for mental illness: impact of a link worker service on admission rates and costs.	2001
Boylen (2020)	Impact of professional interpreters on outcomes for hospitalized children from migrant and refugee families with limited English proficiency: a systematic review.	2020
Bozzola (2020)	Hospitalization for acute cerebellitis in children affected by varicella: how much does it cost?	2020
Brogly (2018)	Neonatal Outcomes in a Medicaid Population With Opioid Dependence.	2018
Burley (2016)	Connecting Patients to Prescription Assistance Programs: Effects on Emergency Department and Hospital Utilization.	2016
Burton (2018)	Statin Use is Not Associated with Future Long-Term Care Admission: Extended Follow-Up of Two Randomised Controlled Trials.	2018
Davis (2022)	A Randomized Controlled Trial Evaluating the Effectiveness of Supported Employment Integrated in Primary Care.	2022
Desai (2016)	Association Between Hospital Penalty Status Under the Hospital Readmission Reduction Program and Readmission Rates for Target and Nontarget Conditions	2016
DeWalt (2004)	Development and pilot testing of a disease management program for low literacy patients with heart failure	2004
DeWalt (2006)	A heart failure self-management program for patients of all literacy levels: a randomized, controlled trial [ISRCTN11535170].	2006
Fahey (2015)	Charitable pharmacy services: Impact on patient-reported hospital use, medication access, and health status.	2015

Forchuk (2008)	Developing and testing an intervention to prevent homelessness among individuals discharged from psychiatric wards to shelters and 'No Fixed Address'	2008
Fors (2016)	Effectiveness of person-centred care after acute coronary syndrome in relation to educational level: Subgroup analysis of a two-armed randomised controlled trial	2016
Frank (2006)	Heat or eat: The low income home energy assistance program and nutritional and health risks among children less than 3 years of age	2006
Fraser (2000)	Comparison of midwifery care to medical care in hospitals in the Quebec pilot projects study: clinical indicators. L'Equipe dEvaluation des Projets-Pilotes Sages-Femmes.	2000
Fretwell (1990)	The Senior Care Study. A controlled trial of a consultative/unit-based geriatric assessment program in acute care	1990
Fuji (2024)	Effect of a Financial Education and Coaching Program for Low-Income, Single Mother Households on Child Health Outcomes	2024
Gaskin (2018)	Racial and Ethnic Composition of Hospitals' Service Areas and the Likelihood of Being Penalized for Excess Readmissions by the Medicare Program	2018
Gate (2016)	Promoting lifestyle behaviour change and well-being in hospital patients: a pilot study of an evidence-based psychological intervention.	2016
Gattis (2004)	Predischarge initiation of carvedilol in patients hospitalized for decompensated heart failure	2004
Gelkopf (2012)	Nonmedication smoking reduction program for inpatients with chronic schizophrenia: a randomized control design study.	2012
Ghahramanlou-Holloway (2012)	Post-Admission Cognitive Therapy: A Brief Intervention for Psychiatric Inpatients Admitted After a Suicide Attempt	2012
Helge (2014)	Street football is a feasible health-enhancing activity for homeless men: Biochemical bone marker profile and balance improved	2014
Hellström (2023)	Predictors of Return to Work for People with Anxiety or Depression Participating in a Randomized Trial Investigating the Effect of a Supported Employment Intervention.	2023
Huckfeldt (2019)	Thirty-Day Postdischarge Mortality Among Black and White Patients 65 Years and Older in the Medicare Hospital Readmissions Reduction Program.	2019
Johnston (2021)	Association of Race and Ethnicity and Medicare Program Type With Ambulatory Care Access and Quality Measures	2021
Justvig (2022)	The Role of Social Determinants of Health in the Use of Telemedicine for Asthma in Children	2022
Kopanitsa (2023)	A systematic scoping review of primary health care service outreach for homeless populations.	2023
Higgins	Leveraging the cigarette purchase task to understand relationships between cumulative vulnerabilities, the relative reinforcing effects of smoking, and response to reduced nicotine content cigarettes.	2
Hoffmann (2014)	Long-Term Effectiveness of Supported Employment: 5-Year Follow-Up of a Randomized Controlled Trial	2014
Lyhne (2022)	Interventions to Prevent Potentially Avoidable Hospitalizations: A Mixed Methods Systematic Review	2022
Mallick (2022)	The impact of co-location employment partnerships within the Australian mental health service and policy context: A systematic review.	2022
Manickas-Hill (2019)	A Review of Bundled Payments in Total Joint Replacement	2019
Moffatt (2023)	Impact of a social prescribing intervention in North East England on adults with type 2 diabetes: the SPRING_NE multimethod study.	2023
Morphew (2013)	Mobile health care operations and return on investment in predominantly underserved children with asthma: the breathmobile program.	2013
Swann	Return on investment of self-management education and home visits for children with asthma	2
Szanton (2021)	CAPABLE program improves disability in multiple randomized trials	2021
Walker (2014)	Pregnancy, prison and perinatal outcomes in New South Wales, Australia: a retrospective cohort study using linked health data.	2014
Wildman (2023)	Impact of a link worker social prescribing intervention on non-elective admitted patient care costs: A quasi-experimental study.	2023
Fenick	A Randomized Controlled Trial of Group Well-Child Care: Improved Attendance and Vaccination Timeliness	2
Abbott (2003)	Hospitalized psychoses after renal transplantation in the United States: incidence, risk factors, and prognosis.	2003
Abel (2018)	Reducing emergency hospital admissions: a population health complex intervention of an enhanced model of primary care and compassionate communities.	2018
Adamson (2016)	Pulmonary Artery Pressure-Guided Heart Failure Management Reduces 30-Day Readmissions.	2016
Alesiani (2014)	Systems Training for Emotional Predictability and Problem Solving (STEPPS): Program efficacy and personality features as predictors of drop-out - An Italian study	2014
Bates	Applying STAAR Interventions in Incremental Bundles: Improving Post-CABG Surgical Patient Care.	2
Auerbach (2002)	Implementation of a voluntary hospitalist service at a community teaching hospital: improved clinical efficiency and patient outcomes.	2002
Axon (2016)	Dual health care system use is associated with higher rates of hospitalization and hospital readmission among veterans with heart failure.	2016

Banerjee (2021)	Association between Medicare's Hospital Readmission Reduction Program and readmission rates across hospitals by medicare bed share.	2021
Barath (2020)	Accountable Care Organizations and Preventable Hospitalizations Among Patients With Depression.	2020
Benjamin-Chung (2020)	Evaluation of a city-wide school-located influenza vaccination program in Oakland, California, with respect to vaccination coverage, school absences, and laboratory-confirmed influenza: A matched cohort study	2020
Benjenk (2020)	Evidence of the Linkage between Hospital-based Care Coordination Strategies and Hospital Overall (Star) Ratings	2020
Benthien (2022)	Proactive Health Support: a randomised controlled trial of telephone-based self-management support for persons at risk of hospital admission	2022
Benthien (2023)	Who benefits from self-management support? Results from a randomized controlled trial	2023
Bhandari (2023)	Interventions to Reduce Hospital Readmissions in Older African Americans: A Systematic Review of Studies Including African American Patients.	2023
Birman-Deych (2006)	Use and effectiveness of warfarin in medicare beneficiaries with atrial fibrillation	2006
Boege (2014)	Effectiveness of hometreatment in light of clinical elements, chances, and limitations	2014
Borup (2019)	Healthcare use before and after changing disability pension policy: a regional Danish cohort study.	2019
Towenshend	BReATHE interventions (Beating Regional Asthma Through Health Education)-an innovative approach to children's asthma care in the North East and North Cumbria, UK: an interventional study	2
Brooker (2007)	Admission decisions following contact with an emergency mental health assessment and intervention service.	2007
Butz (2010)	Influence of Caregiver and Provider Communication on Symptom Days and Medication Use for Inner-City Children With Asthma	2010
Capomolla (2004)	Heart failure case disease management program: a pilot study of home telemonitoring versus usual care	2004
Catov (2005)	Asthma home teaching: two evaluation approaches	2005
Chaiyachati (2018)	Changes to Racial Disparities in Readmission Rates After Medicare's Hospital Readmissions Reduction Program Within Safety-Net and Non-Safety-Net Hospitals.	2018
Chambers (2023)	Reducing unplanned hospital admissions from care homes: a systematic review.	2023
Charles (2020)	Improving transitions from acute care to home among complex older adults using the LACE Index and care coordination.	2020
Chartrand (2023)	Patient- and family-centred care transition interventions for adults: a systematic review and meta-analysis of RCTs.	2023
Chen (2019)	Hospital Readmissions Reduction Program: Intended and Unintended Effects	2019
Chirila (2017)	Health care resource use analysis of paliperidone palmitate 3 month injection from two phase 3 clinical trials	2017
Chovanec (2021)	Association of Discharge Disposition with Outcomes.	2021
Clar (2003)	Routine hospital admission versus out-patient or home care in children at diagnosis of type 1 diabetes mellitus	2003
Cleland (2005)	Noninvasive home telemonitoring for patients with heart failure at high risk of recurrent admission and death: the Trans-European Network-Home-Care Management System (TEN-HMS) study	2005
Clement (2018)	Urban-Rural Differences in Skilled Nursing Facility Rehospitalization Rates.	2018
Coombes (2023)	Discharge interventions for First Nations people with a chronic condition or injury: a systematic review	2023
Dai (2022)	Family Practices in Transforming Clinical Practice Initiative Showed No Changes in Medicare Costs or Utilization	2022
Dale (2003)	An evaluation of the west Surrey telemedicine monitoring project	2003
Davis (2015)	Innovative Care Models for High-Cost Medicare Beneficiaries: Delivery System and Payment Reform to Accelerate Adoption	2015
de Oliveira (2023)	Psychoeducational Intervention for Reducing Heart Failure Patients' Rehospitalizations and Promoting Their Quality of Life and Posttraumatic Growth at the 1-Year Follow-Up: A Randomized Clinical Trial.	2023
Deek (2016)	Family-centred approaches to healthcare interventions in chronic diseases in adults: a quantitative systematic review	2016
Rotenstein	Development of a Primary Care Transitions Clinic in an Academic Medical Center	2
Doherty (2022)	Minding the gap-an examination of a pharmacist case management medicines optimisation intervention for older people in intermediate care settings	2022
Dols (2020)	Relationship of Nurse-Led Education Interventions to Liver Transplant Early Readmission.	2020
Duminy (2022)	Complex community health and social care interventions - Which features lead to reductions in hospitalizations for ambulatory care sensitive conditions? A systematic literature review.	2022

Dummit (2016)	Association Between Hospital Participation in a Medicare Bundled Payment Initiative and Payments and Quality Outcomes for Lower Extremity Joint Replacement Episodes.	2016
Dunleavy (2019)	Medical, Mental Health, and Social Service Linkage Predicts Better HIV Outcomes: A Network Analytic Approach.	2019
Dunn (2021)	The impact of community nurse-led interventions on the need for hospital use among older adults: An integrative review.	2021
Dye (2018)	Improving Chronic Disease Self-Management by Older Home Health Patients through Community Health Coaching.	2018
Edwards (2017)	Preventing Hospitalization with Veterans Affairs Home-Based Primary Care: Which Individuals Benefit Most?	2017
Eisenstein (2002)	Vasopeptidase inhibitor reduces inhospital costs for patients with congestive heart failure: results from the IMPRESS trial. Inhibition of Metallo Protease by BMS-186716 in a Randomized Exercise and Symptoms Study in Subjects With Heart Failure	2002
Eisenstein (2009)	Long-term clinical and economic analysis of the Endeavor drug-eluting stent versus the Driver bare-metal stent: 4-year results from the ENDEAVOR II trial (Randomized Controlled Trial to Evaluate the Safety and Efficacy of the Medtronic AVE ABT-578 Eluting Driver Coronary Stent in De Novo Native Coronary Artery Lesions).	2009
El-Gamil (2017)	What is the best setting for receiving dialysis vascular access repair and maintenance services?.	2017
Ell (2010)	Collaborative Depression Treatment in Older and Younger Adults With Physical Illness: Pooled Comparative Analysis of Three Randomized Clinical Trials	2010
Epstein (2001)	The role of public clinics in preventable hospitalizations among vulnerable populations.	2001
Esslinger (2014)	Exploratory analysis of the relationship between home health agency engagement in a national campaign and reduction in acute care hospitalization in US home care patients	2014
Wilcox	Evaluation of a Hospital: Community Partnership to Reduce 30-Day Readmissions.	2
Finlayson (2018)	Transitional care interventions reduce unplanned hospital readmissions in high-risk older adults	2018
Fish-Trotter (2018)	Design and rationale of a randomized trial: Using short stay units instead of routine admission to improve patient centered health outcomes for acute heart failure patients (SSU-AHF).	2018
Fitchett (2018)	Effects of empagliflozin on risk for cardiovascular death and heart failure hospitalization across the spectrum of heart failure risk in the EMPA-REG OUTCOME® trial	2018
Fitzpatrick (2021)	Palivizumab's real-world effectiveness: a population-based study in Ontario, Canada, 1993-2017	2021
Fleming (1997)	Brief physician advice for problem alcohol drinkers - A randomized controlled trial in community-based primary care practices	1997
Fleming (2018)	Early Ambulation Among Hospitalized Heart Failure Patients Is Associated With Reduced Length of Stay and 30-Day Readmissions	2018
Forbes (2006)	Evaluation of a MS specialist nurse programme.	2006
Fornaro (2022)	Homelessness and health-related outcomes: an umbrella review of observational studies and randomized controlled trials.	2022
Froelicher (2004)	Women's Initiative for Nonsmoking-VII: evaluation of health service utilization and costs among women smokers with cardiovascular disease.	2004
Fry (2018)	Comparison of Risk-Adjusted Outcomes in Medicare Open versus Laparoscopic Cholecystectomy.	2018
Fung (2013)	Adverse clinical events among medicare beneficiaries using antipsychotic drugs: linking health insurance benefits and clinical needs.	2013
Gaalema (2019)	Financial Incentives to Increase Cardiac Rehabilitation Participation Among Low-Socioeconomic Status Patients A Randomized Clinical Trial	2019
Gaillard (2022)	Does integrated care mean fewer hospitalizations? An evaluation of a French field experiment	2022
Ganapathy (2017)	Continuing care with nebulized bronchodilators after hospital discharge and impact on readmissions: analysis of medicare COPD beneficiaries receiving arformoterol vs nebulized short-acting agents	2017
George (1999)	A comprehensive educational program improves clinical outcome measures in inner-city patients with asthma	1999
Ghani (2016)	Needle disinfectant technique during prostate biopsy is associated with less infection-related hospitalization: Results from a surgical collaborative	2016
Gheorghiade (2013)	Effect of oral digoxin in high-risk heart failure patients: a pre-specified subgroup analysis of the DIG trial.	2013
Gilman (2014)	Effectiveness of a post-emergency department automated telephone call on follow-up appointment compliance and association of compliance with subsequent hospitalization	2014
Goff (2017)	Effects of an Enhanced Primary Care Program on Diabetes Outcomes.	2017
Golberstein (2015)	Effect of the Affordable Care Act's Young Adult Insurance Expansions on Hospital-Based Mental Health Care	2015
Gomis-Pastor (2023)	Does an eHealth Intervention Reduce Complications and Healthcare Resources? A mHeart Single-Center Randomized-Controlled Trial	2023
Gorostiza (2021)	Dynamic evaluation of the comparative effectiveness of an integrated program for heart failure care	2021
Gruneir (2007)	Hospitalization of nursing home residents with cognitive impairments: the influence of organizational features and state policies.	2007

Guo (2001)	Assessing the impact of community-based mobile crisis services on preventing hospitalization.	2001
Gupta (2018)	The Hospital Readmissions Reduction Program: Evidence for Harm.	2018
Hahn (2014)	Fewer Hospitalizations for Chronic Obstructive Pulmonary Disease in Communities With Smoke-Free Public Policies	2014
Hallgren (2013)	The Swedish six-community alcohol and drug prevention trial: effects on youth drinking	2013
Harrop (2024)	Effects of a culturally informed model of care for Aboriginal and Torres Strait Islander patients with acute coronary syndrome in a tertiary hospital in Australia: a pre-post, quasi-experimental, interventional study.	2024
Hatfield (2016)	Survival and Toxicity After Cisplatin Plus Etoposide Versus Carboplatin Plus Etoposide for Extensive-Stage Small-Cell Lung Cancer in Elderly Patients.	2016
Havers (2016)	Case-Control Study of Vaccine Effectiveness in Preventing Laboratory-Confirmed Influenza Hospitalizations in Older Adults, United States, 2010-2011	2016
Hollinghurst (2022)	Do home adaptation interventions help to reduce emergency fall admissions? A national longitudinal data-linkage study of 657,536 older adults living in Wales (UK) between 2010 and 2017.	2022
Horner (2016)	Enhancing Asthma Self-Management in Rural School-Aged Children: A Randomized Controlled Trial	2016
Ibrahim (2018)	Emergency Surgery for Medicare Beneficiaries Admitted to Critical Access Hospitals.	2018
Inampudi (2014)	Spironolactone use and higher hospital readmission for Medicare beneficiaries with heart failure, left ventricular ejection fraction <45%, and estimated glomerular filtration rate <45 ml/min/1.73 m(2.).	2014
Ingber (2017)	Initiative To Reduce Avoidable Hospitalizations Among Nursing Facility Residents Shows Promising Results	2017
Inohara (2018)	Association of Renin-Angiotensin Inhibitor Treatment with Mortality and Heart Failure Readmission in Patients with Transcatheter Aortic Valve Replacement	2018
Duckett	Investigating a Multistakeholder Alliance Approach to Reducing Hospital Readmissions.	2
IsHak (2024)	Comparative Effectiveness of Psychotherapy vs Antidepressants for Depression in Heart Failure: A Randomized Clinical Trial.	2024
Jäckel (2017)	Effects of Sustained Competitive Employment on Psychiatric Hospitalizations and Quality of Life	2017
Janevic (2022)	Analysis of State Medicaid Expansion and Access to Timely Prenatal Care Among Women Who Were Immigrant vs US Born.	2022
Jennings (2019)	Health Care Utilization and Cost Outcomes of a Comprehensive Dementia Care Program for Medicare Beneficiaries	2019
Jha (2007)	Performance measures, vaccinations, and pneumonia rates among high-risk patients in Veterans Administration health care.	2007
Joo (2014)	Community-Based Case Management, Hospital Utilization, and Patient-Focused Outcomes in Medicare Beneficiaries.	2014
Kane (2017)	Effects of an Intervention to Reduce Hospitalizations From Nursing Homes: A Randomized Implementation Trial of the INTERACT Program.	2017
Khazanie (2014)	Trends in the use and outcomes of ventricular assist devices among medicare beneficiaries, 2006 through 2011.	2014
Kilburn (2017)	Home Visiting and Use of Infant Health Care: A Randomized Clinical Trial.	2017
Kinsey (2023)	Impact of interventions to improve recovery of older adults following planned hospital admission on quality-of-life following discharge: linked-evidence synthesis.	2023
Klug (2014)	North Dakota assistance program for dementia caregivers lowered utilization, produced savings, and increased empowerment.	2014
Koniak-Griffin (2002)	Public health nursing care for adolescent mothers: Impact on infant health and selected maternal outcomes at 1 year postbirth	2002
Kosiborod (2005)	Anemia and outcomes in patients with heart failure: a study from the National Heart Care Project.	2005
Kronman (2008)	Can primary care visits reduce hospital utilization among Medicare beneficiaries at the end of life?.	2008
Krska (2001)	Pharmacist-led medication review in patients over 65: a randomized, controlled trial in primary care	2001
Kuo (2013)	Association between proportion of provider clinical effort in nursing homes and potentially avoidable hospitalizations and medical costs of nursing home residents.	2013
Kuo (2015)	Potentially Preventable Hospitalizations in Medicare Patients With Diabetes: A Comparison of Primary Care Provided by Nurse Practitioners Versus Physicians.	2015
Leary (2023)	Building an inpatient addiction medicine consult service in Sudbury, Canada: preliminary data and lessons learned in the era of COVID-19	2023
Leckcivilize (2021)	Impact of an anticipatory care planning intervention on unscheduled acute hospital care using difference-in-difference analysis.	2021
Lee (2018)	Spillover effects of the hospital readmission reduction program on radical cystectomy readmissions	2018
Li (2018)	Does Medicare Advantage Reduce Racial Disparity in 30-Day Rehospitalization for Medicare Beneficiaries?.	2018
Lichtman (2009)	Stroke Patient Outcomes in US Hospitals Before the Start of the Joint Commission Primary Stroke Center Certification Program	2009

Lindstroem (2021)	The effect of the employment of experienced physicians in the Emergency Department on quality of care and equality-a quasi-experimental retrospective cohort study	2021
Lutfiyya (2017)	Does primary care diabetes management provided to Medicare patients differ between primary care physicians and nurse practitioners?	2017
Mann (2021)	Impact of an integrated community-based model of care for older people with complex conditions on hospital emergency presentations and admissions: a step-wedged cluster randomized trial.	2021
Marafino (2021)	Evaluation of an intervention targeted with predictive analytics to prevent readmissions in an integrated health system: observational study.	2021
Masoudi (2014)	Comparative effectiveness of cardiac resynchronization therapy with an implantable cardioverter-defibrillator versus defibrillator therapy alone: a cohort study.	2014
McDermott (2001)	Improving diabetes care in the primary healthcare setting: a randomised cluster trial in remote Indigenous communities	2001
McWilliams (2013)	Changes in Health Care Spending and Quality for Medicare Beneficiaries Associated With a Commercial ACO Contract	2013
Miller (2017)	Management of Heart Failure in a Rural Community.	2017
Momesso (2023)	Effect of removing the 4-hour access standard in the ED: a retrospective observational study.	2023
Morello (2016)	A Telephone Support Program to Reduce Costs and Hospital Admissions for Patients at Risk of Readmissions: Lessons from an Evaluation of a Complex Health Intervention.	2016
Moreno (2021)	Connecting Provider to home: A home-based social intervention program for older adults	2021
Murphy (2019)	2018 John Charnley Award: Analysis of US Hip Replacement Bundled Payments: Physician-initiated Episodes Outperform Hospital-initiated Episodes.	2019
Musich (2014)	CLINICAL. Personalized Preventive Care Reduces Healthcare Expenditures Among Medicare Advantage Beneficiaries.	2014
Nguyen (2023)	Effect of the population health inpatient Medicare Advantage pharmacist intervention on hospital readmissions: A quasi-experimental controlled study.	2023
Nguyen (2023)	Thirty-Day Unplanned Readmissions Following Elective and Acute Percutaneous Coronary Intervention	2023
North (2019)	Design, Implementation, and Assessment of a Public Comprehensive Specialty Care Program for Early Psychosis	2019
Oliva (2008)	The impact of RN case management on inpatient and ED utilization in a chronically ill, older adult, community-dwelling population.	2008
Ong (2017)	A Community-Partnered, Participatory, Cluster-Randomized Study of Depression Care Quality Improvement: Three-Year Outcomes	2017
Orzol (2018)	The Impact of a Health Information Technology-Focused Patient-centered Medical Neighborhood Program Among Medicare Beneficiaries in Primary Care Practices: The Effect on Patient Outcomes and Spending.	2018
Pandolfi (2017)	Associations between nursing home performance and hospital 30-day readmissions for acute myocardial infarction, heart failure and pneumonia at the healthcare community level in the United States.	2017
Peikes (2009)	Effects of Care Coordination on Hospitalization, Quality of Care, and Health Care Expenditures Among Medicare Beneficiaries 15 Randomized Trials	2009
Portnoy (2006)	Utilization patterns in an asthma intervention.	2006
Reddy (2020)	Association of High-Cost Health Care Utilization With Longitudinal Changes in Patient-Centered Medical Home Implementation.	2020
Riera-Molist (2023)	A Brief Psychoeducation Intervention to Prevent Rehospitalization in Severe Mental Disorder Inpatients.	2023
Riley (2015)	Program evaluation of remote heart failure monitoring: healthcare utilization analysis in a rural regional medical center.	2015
Roberts (2018)	The Value-Based Payment Modifier: Program Outcomes and Implications for Disparities.	2018
Roy (2023)	Association Between Hospital Participation in Value-Based Programs and Timely Initiation of Post-Acute Home Health Care, Functional Recovery, and Hospital Readmission After Joint Replacement.	2023
Rymer (2018)	Advanced Practice Provider Versus Physician-Only Outpatient Follow-Up After Acute Myocardial Infarction.	2018
Saleh (2012)	An effectiveness and cost-benefit analysis of a hospital-based discharge transition program for elderly Medicare recipients.	2012
Sandhu (2019)	Comparison of the change in heart failure readmission and mortality rates between hospitals subject to hospital readmission reduction program penalties and critical access hospitals.	2019
Schermerhorn (2008)	Endovascular vs. open repair of abdominal aortic aneurysms in the medicare population	2008
Schley (2008)	Early intervention with difficult to engage, 'high-risk' youth: evaluating an intensive outreach approach in youth mental health.	2008
Schmidt-Kraepelin (2009)	Prevention of rehospitalization in schizophrenia: results of an integrated care project in Germany.	2009
Schraeder (2001)	The effects of a collaborative model of primary care on the mortality and hospital use of community-dwelling older adults.	2001
Segal (2016)	Economic evaluation of Indigenous health worker management of poorly controlled type 2 diabetes in north Queensland.	2016

Shafi (2017)	Antihypertensive medications and risk of death and hospitalizations in US hemodialysis patients Evidence from a cohort study to inform hypertension treatment practices	2017
Shah (2016)	COPD Readmissions: Addressing COPD in the Era of Value-based Health Care.	2016
Shin (2013)	Effect of antihypertensive medication adherence on hospitalization for cardiovascular disease and mortality in hypertensive patients	2013
Sibille (2023)	Benzodiazepine Receptor Agonists Use and Cessation Among Multimorbid Older Adults with Polypharmacy: Secondary Analysis from the OPERAM Trial.	2023
Smith (2016)	Treatment patterns, overall survival, healthcare resource use and costs in elderly Medicare beneficiaries with chronic myeloid leukemia using second-generation tyrosine kinase inhibitors as second-line therapy	2016
Smith (2022)	Impactability Modeling for Reducing Medicare Accountable Care Organization Payments and Hospital Events in High-Need High-Cost Patients: Longitudinal Cohort Study	2022
Som (2017)	Improving Dialysis Adherence for High Risk Patients Using Automated Messaging: proof of Concept	2017
Sommer (2011)	Children's Hospital Boston Community Asthma Initiative: Partnerships and Outcomes Advance Policy Change	2011
Spangler (2023)	The Impact of the Swedish Care Coordination Act on Hospital Readmission and Length-of-Stay among Multi- Morbid Elderly Patients: A Controlled Interrupted Time Series Analysis	2023
Spitzer (2020)	A geographic analysis of racial disparities in use of pulmonary rehabilitation after hospitalization for COPD exacerbation	2020
Stas (2023)	Implications of a Reduced Length of Postpartum Hospital Stay on Maternal and Neonatal Readmissions, an Observational Study.	2023
Stergiopoulos (2015)	Effectiveness of Housing First with Intensive Case Management in an Ethnically Diverse Sample of Homeless Adults with Mental Illness: A Randomized Controlled Trial	2015
Stuart (2010)	Impact of maintenance therapy on hospitalization and expenditures for Medicare beneficiaries with chronic obstructive pulmonary disease.	2010
Betancourt	Substance Use Relapse Among Veterans at Termination of Treatment for Substance Use Disorders.	2
Swankoski (2023)	Intensive care management for high-risk veterans in a patient-centered medical home - do some veterans benefit more than others?.	2023
Swanson (2022)	Association between primary care appointment lengths and subsequent ambulatory reassessment, emergency department care, and hospitalization: a cohort study	2022
Takaku (2016)	Effects of reduced cost-sharing on children's health: Evidence from Japan	2016
Tantipinichwong (2017)	Impact on 30-day hospital readmissions of post-discharge medication reconciliation in a medicare advantage patient population	2017
Ten (2019)	The Impact of a Heart Failure Management Program in a Medicare Advantage Population	2019
Thomas (2009)	High-dose inhaled corticosteroids versus add-on long-acting beta-agonists in asthma: an observational study.	2009
Thompson (2018)	Community Navigators Reduce Hospital Utilization in Super-Utilizers.	2018
Timbie (2017)	Implementation of Medical Homes in Federally Qualified Health Centers.	2017
Tran (2024)	The Australian Health Care Homes trial: quality of care and patient outcomes. A propensity score-matched cohort study	2024
Unoki (2024)	Exploring the influence of a financial incentive scheme on early mobilization and rehabilitation in ICU patients: an interrupted time-series analysis.	2024
Unruh (2017)	Hospitalization event notifications and reductions in readmissions of Medicare fee-for-service beneficiaries in the Bronx, New York	2017
Unruh (2018)	Hospital participation in Meaningful Use and racial disparities in readmissions.	2018
van Loon-van (2021)	Telephone follow-up to reduce unplanned hospital returns for older emergency department patients: A randomized trial.	2021
Vergara (2021)	Predictors for Telephone Outreach Post-hospital Discharge.	2021
Vohr (2017)	Impact of a Transition Home Program on Rehospitalization Rates of Preterm Infants.	2017
Wadhera (2019)	Association of State Medicaid Expansion With Quality of Care and Outcomes for Low-Income Patients Hospitalized With Acute Myocardial Infarction.	2019
Wang (2010)	Impact of Drug Cost Sharing on Service Use and Adverse Clinical Outcomes in Elderly Receiving Antidepressants	2010
Welch (2009)	Assessment of the impact of medication therapy management delivered to home-based Medicare beneficiaries	2009
Whitcomb (2019)	Association of Decision Support for Hospital Discharge Disposition With Outcomes.	2019
Wong (2018)	Effects of the VA patient centered medical home initiative on healthcare utilization: Results after four years	2018
Xu (2022)	The impact of community nursing program on healthcare utilization: A program evaluation.	2022
Xu (2022)	Does the abolition of copayment increase ambulatory care utilization?: a quasi-experimental study in Germany	2022
Zabawa (2018)	Thirty-day rehospitalizations among elderly patients with acute myocardial infarction Impact of postdischarge ambulatory care	2018

Zillich (2013)	A randomized, controlled pragmatic trial of telephonic medication therapy management to reduce hospitalization in home health patients	2013
Zillich (2014)	A Randomized, Controlled Pragmatic Trial of Telephonic Medication Therapy Management to Reduce Hospitalization in Home Health Patients	2014

Exclude on study period, n=4

Ahring (1992)	Telephone modem access improves diabetes control in those with insulin-requiring diabetes	1992
Gadomski (1998)	Impact of a Medicaid primary care provider and preventive care on pediatric hospitalization	1998
GAZIANO (1994)	IS IT TIME TO REASSESS THE RISK FOR THE GROWTH-RETARDED FETUS WITH NORMAL DOPPLER VELOCIMETRY OF THE UMBILICAL ARTERY	1994
Geddes (1994)	Prediction of outcome following a first episode of schizophrenia. A follow-up study of Northwick Park first episode study subjects	1994

Exclude on study intervention universal to all population, n=36

Almquist (2022)	The impact of an unemployment insurance reform on incidence rates of hospitalisation due to alcohol-related disorders: a quasi-experimental study of heterogeneous effects across ethnic background, educational level, employment status, and sex in Sweden.	2022
Elmer (2014)	Summary of: An alternative marker for the effectiveness of water fluoridation: hospital extraction rates for dental decay, a two-region study	2014
Grotting (2020)	Health effects of retirement: evidence from survey and register data	2020
Herttua (2015)	Educational inequalities in hospitalization attributable to alcohol: a population-based longitudinal study of changes during the period 2000-07.	2015
Mackay (2021)	Associations between smoke-free vehicle legislation and childhood admissions to hospital for asthma in Scotland: an interrupted time-series analysis of whole-population data	2021
Millett (2013)	Hospital Admissions for Childhood Asthma After Smoke-Free Legislation in England	2013
Petousis (2019)	Pneumococcal Conjugate Vaccines Turning the Tide on Inequity: A Retrospective Cohort Study of New Zealand Children Born 2006–2015.	2019
Rezansoff (2015)	Beyond recidivism: changes in health and social service involvement following exposure to drug treatment court.	2015
Rose (2021)	Impact of local air quality management policies on emergency hospitalisations for respiratory conditions in the North West Coast region of England: a longitudinal controlled ecological study	2021
Turner (2020)	Associations between a smoke-free homes intervention and childhood admissions to hospital in Scotland: an interrupted time-series analysis of whole-population data.	2020
Wyper (2023)	Evaluating the impact of alcohol minimum unit pricing on deaths and hospitalisations in Scotland: a controlled interrupted time series study.	2023
Zhao (2017)	The impacts of minimum alcohol pricing on alcohol attributable morbidity in regions of British Colombia, Canada with low, medium and high mean family income	2017
Pressley (2009)	Motor Vehicle Occupant Injury and Related Hospital Expenditures in Children Aged 3 Years to 8 Years Covered Versus Uncovered by Booster Seat Legislation	2009
Madden (2002)	Effects of a law against early postpartum discharge on newborn follow-up, adverse events, and HMO expenditures.	2002
Capdevila (2023)	Population-based evaluation of the impact of socioeconomic status on clinical outcomes in patients with heart failure in integrated care settings.	2023
Gosselin (2016)	Effectiveness of rotavirus vaccine in preventing severe gastroenteritis in young children according to socioeconomic status	2016
Gosselin (2016)	Trends in severe gastroenteritis among young children according to socio-economic characteristics before and after implementation of a rotavirus vaccination program in Quebec	2016
Hungerford (2018)	Rotavirus vaccine impact and socioeconomic deprivation: An interrupted time-series analysis of gastrointestinal disease outcomes across primary and secondary care in the UK	2018
Piroddi (2022)	The impact of an integrated care intervention on mortality and unplanned hospital admissions in a disadvantaged community in England: A difference-in-differences study.	2022
Angraal (2018)	Trends in 30-Day Readmission Rates for Medicare and Non-Medicare Patients in the Era of the Affordable Care Act.	2018
Bell (2016)	Effect of Pharmacist Counseling Intervention on Health Care Utilization Following Hospital Discharge: a Randomized Control Trial	2016
Blanchard (2018)	State variation in opioid treatment policies and opioid-related hospital readmissions.	2018
Cheon (2020)	An exploration of community partnerships, safety-net hospitals, and readmission rates.	2020
Chou (2021)	Impact of High-Deductible Health Plans on Emergency Department Patients With Nonspecific Chest Pain and Their Subsequent Care	2021
Colla (2012)	Spending Differences Associated With the Medicare Physician Group Practice Demonstration	2012

Connell (2020)	Health Care Reform, Length of Stay, and Readmissions for Child Mental Health Hospitalizations	2020
DeWalt (2012)	Multisite Randomized Trial of a Single-Session Versus Multisession Literacy-Sensitive Self-Care Intervention for Patients With Heart Failure	2012
Lu (2016)	Reducing excess readmissions: promising effect of hospital readmissions reduction program in US hospitals.	2016
Pimentel (2017)	Impact of Health Policy Changes on Emergency Medicine in Maryland Stratified by Socioeconomic Status.	2017
Murty	Primary Care Availability, Safety Net Clinics, and Health Insurance Coverage.	2
Salerno (2017)	Trends in readmission rates for safety net hospitals and non-safety net hospitals in the era of the US Hospital Readmission Reduction Program: A retrospective time series analysis using Medicare administrative claims data from 2008 to 2015	2017
Sankaran (2019)	Changes in hospital safety following penalties in the US Hospital Acquired Condition Reduction Program: retrospective cohort study.	2019
Wharam (2018)	Effect of High-deductible insurance on high-acuity outcomes in diabetes: A natural experiment for translation in diabetes (NEXT-D) study	2018
Garbutt (2015)	A cluster-randomized trial shows telephone peer coaching for parents reduces children's asthma morbidity	2015
Meyers (2019)	Association of Team-Based Primary Care With Health Care Utilization and Costs Among Chronically Ill Patients.	2019
Soto-Gordoa (2019)	Gender and socioeconomic inequalities in the implementation of the Basque programme for multimorbid patients.	2019

Exclude on country, n=117

de Cuba (2023)	Child Care Feeding Programs Associated With Food Security and Health for Young Children From Families With Low Incomes	2023
Pollack (2023)	Using the Moving to Opportunity Experiment to Investigate the Long-Term Impact of Neighborhoods on Healthcare Use by Specific Clinical Conditions and Type of Service	2023
Watkins (2001)	Changes in mental health and service use after termination of SSI benefits	2001
Bischoff (2013)	Advance care planning and the quality of end-of-life care in older adults.	2013
Blumenthal (2017)	Association Between Treatment by Locum Tenens Internal Medicine Physicians and 30-Day Mortality Among Hospitalized Medicare Beneficiaries.	2017
Brown (2012)	Six Features Of Medicare Coordinated Care Demonstration Programs That Cut Hospital Admissions Of High-Risk Patients	2012
Castro (2003)	Asthma intervention program prevents readmissions in high healthcare users	2003
Chan (2023)	Ambulatory Intensive Care for Medically Complex Patients at a Health Care Clinic for Individuals Experiencing Homelessness: The SUMMIT Randomized Clinical Trial.	2023
Gallo (2023)	Mi Puente (My Bridge) Care Transitions Program for Hispanic/Latino Adults with Multimorbidity: Results of a Randomized Controlled Trial.	2023
Guo (2005)	Impact of school-based health centers on children with asthma	2005
Harrison-Long (2023)	The impact of the baby friendly hospital initiative on healthcare utilization among newborns insured by Medicaid in Delaware	2023
Heisler (2022)	Impact on Health Care Utilization and Costs of a Medicaid Community Health Worker Program in Detroit, 2018-2020: A Randomized Program Evaluation.	2022
Jackson (2013)	Transitional care cut hospital readmissions for North Carolina Medicaid patients with complex chronic conditions.	2013
Lavallee (2023)	Supplemental Nutrition Assistance Program Emergency Allotments and Food Security, Hospitalizations, and Hospital Capacity.	2023
Lob (2000)	Case management: a controlled evaluation of persons with diabetes.	2000
Mainardi (2023)	Reducing asthma exacerbations in vulnerable children through a medical-legal partnership.	2023
Mattison (2023)	Effectiveness of a community-driven, asthma intervention: project asthma in-home response	2023
Msw (2023)	A descriptive study of screening and navigation on health-related social needs in a safety-net hospital emergency department.	2023
Pantell (2022)	Association of 2 Social Needs Interventions With Child Emergency Department Use and Hospitalizations A Secondary Analysis of a Randomized Clinical Trial	2022
Park-Clinton (2023)	A Targeted Discharge Planning for High-Risk Readmissions: Focus on Patients and Caregivers.	2023
Quinton (2023)	Differential Impact of a Plan-Led Standardized Complex Care Management Intervention on Subgroups of High-Cost High-Need Medicaid Patients	2023
Rabito (2017)	A single intervention for cockroach control reduces cockroach exposure and asthma morbidity in children	2017
Robinson (2008)	The impact of literacy enhancement on asthma-related outcomes among underserved children.	2008

Roth (2023)	Evaluation of an Integrated Intervention to Address Clinical Care and Social Needs Among Patients with Type 2 Diabetes.	2023
Sabbatini (2022)	Impact of a statewide Emergency Department Information Exchange on health care use and expenditures	2022
Smith (2023)	The impacts of the 340B Program on health care quality for low-income patients.	2023
Spoelstra (2022)	Results of a multi-site pragmatic hybrid type 3 cluster randomized trial comparing level of facilitation while implementing an intervention in community-dwelling disabled and older adults in a Medicaid waiver	2022
Steinman (2023)	Can a Home-Based Collaborative Care Model Reduce Health Services Utilization for Older Medicaid Beneficiaries Living with Depression and Co-occurring Chronic Conditions? A Quasi-experimental Study.	2023
Tavares (2023)	The effect of the right care, right place, right time (R3) initiative on Medicare health service use among older affordable housing residents	2023
Yang (2022)	Did the Hospital Readmissions Reduction Program Reduce Readmissions without Hurting Patient Outcomes at High Dual-Proportion Hospitals Prior to Stratification?.	2022
Zhao (2024)	Evaluating the impact of the Medicaid expansion program on diabetes hospitalization.	2024
Allaire (2023)	Does access to free medication reduce health system costs? An evaluation of the Dispensary of Hope program	2023
Aparasu (2014)	Risk of hospitalization and use of first- versus second-generation antipsychotics among nursing home residents.	2014
Apter (2020)	Patient Advocates for Low-Income Adults with Moderate to Severe Asthma: A Randomized Clinical Trial	2020
Balamurugan (2006)	Diabetes self-management education program for medicaid recipients - A continuous quality improvement process	2006
Bera (2014)	Hospitalization resource utilization and costs among Medicaid insured patients with schizophrenia with different treatment durations of long-acting injectable antipsychotic therapy.	2014
Bollinger (2010)	The Breathmobile program: a good investment for underserved children with asthma	2010
Brown (2020)	Impact of the Affordable Care Act Medicaid Expansion on Access to Care and Hospitalization Charges for Lupus Patients.	2020
Carter (2021)	Effect of Community Health Workers on 30-Day Hospital Readmissions in an Accountable Care Organization Population A Randomized Clinical Trial	2021
Castellanos (2016)	Home Monitoring Program Reduces Mortality in High-Risk Sociodemographic Single-Ventricle Patients	2016
Chung (2014)	Impact of a Clinical Pharmacy Program on Changes in Hemoglobin A1c, Diabetes-Related Hospitalizations, and Diabetes-Related Emergency Department Visits for Patients with Diabetes in an Underserved Population	2014
Davidson (2003)	Evaluation of access, a primary care program for indigent patients: Inpatient and emergency room utilization	2003
Dayal (2019)	Hospital Utilization Among Rural Children Served by Pediatric Neurology Telemedicine Clinics.	2019
de la Vega (2023)	A Pharmacy Liaison-Patient Navigation Intervention to Reduce Inpatient and Emergency Department Utilization Among Primary Care Patients in a Medicaid Accountable Care Organization: A Nonrandomized Controlled Trial	2023
Dreisbach (2023)	Improving childhood asthma outcomes in East Harlem: the East Harlem Asthma Center of Excellence's Asthma Counselor Program.	2023
Duggan (2004)	Randomized trial of a statewide home visiting program: impact in preventing child abuse and neglect	2004
Duru (2020)	Evaluation of a National Care Coordination Program to Reduce Utilization Among High-cost, High-need Medicaid Beneficiaries With Diabetes	2020
Dush (2001)	Reducing psychiatric hospital use of the rural poor through intensive transitional acute care.	2001
Eakin (2012)	Asthma in Head Start children: Effects of the Breathmobile program and family communication on asthma outcomes	2012
Edge (2022)	Breaking the Cycle Care Coordination Interventions and Sickle Cell Readmissions	2022
Pantell	Effects of In-Person Navigation to Address Family Social Needs on Child Health Care Utilization: A Randomized Clinical Trial.	2
Englander (2014)	The care transitions innovation (C-train) for socioeconomically disadvantaged adults, results of a clustered randomized controlled trial	2014
Fine (2023)	Health Care Utilization among Homeless-Experienced Adults Who Were Seen by a Mobile Addiction Health Clinic in Boston, Massachusetts: A Quasi-Experimental Study.	2023
Fritz (2022)	Association of Race and Neighborhood Disadvantage with Patient Engagement in a Home-Based COVID-19 Remote Monitoring Program	2022
Gill (2003)	Does having an outpatient visit after hospital discharge reduce the likelihood of readmission?.	2003
Goldman (2014)	Support from hospital to home for elders: a randomized trial.	2014
Gurewich (2011)	Comparative performance of community health centers and other usual sources of primary care.	2011
Healy-Collier (2016)	Medicaid managed care reduces readmissions for youths with type 1 diabetes.	2016
Hefei (2019)	Prescription Drug Monitoring Program Mandates: Impact On Opioid Prescribing And Related Hospital Use.	2019
Houck (2006)	Asthma prevention in urbanites.	2006

Karnick (2007)	The pediatric asthma intervention: A comprehensive cost-effective approach to asthma management in a disadvantaged inner-city community	2007
Kelley (2020)	PATIENT NAVIGATION TO REDUCE EMERGENCY DEPARTMENT (ED) UTILIZATION AMONG MEDICAID INSURED, FREQUENT ED USERS: A RANDOMIZED CONTROLLED TRIAL	2020
Kelly (2000)	Outcomes evaluation of a comprehensive intervention program for asthmatic children enrolled in Medicaid	2000
Kercsmar (2017)	Association of an Asthma Improvement Collaborative With Health Care Utilization in Medicaid-Insured Pediatric Patients in an Urban Community.	2017
Klinnert (2005)	Short-term impact of a randomized multifaceted intervention for wheezing infants in low-income families	2005
Lu (2011)	Association Between Prior Authorization for Medications and Health Service Use by Medicaid Patients With Bipolar Disorder	2011
Mackinney (2013)	Does providing care for uninsured patients decrease emergency room visits and hospitalizations?.	2013
Matone (2012)	Emergency department visits and hospitalizations for injuries among infants and children following statewide implementation of a home visitation model	2012
Murray (2007)	Pharmacist intervention to improve medication adherence in heart failure: a randomized trial	2007
Nelson (2011)	A randomized controlled trial of parental asthma coaching to improve outcomes among urban minority children.	2011
Nelson (2021)	Environmental Health Consults in Children Hospitalized with Respiratory Infections	2021
Ni (2017)	Impact of a pharmacy-based transitional care program on hospital readmissions.	2017
Rothkopf (2011)	Medicaid patients seen at federally qualified health centers use hospital services less than those seen by private providers.	2011
Saloner (2020)	Specialty Substance Use Disorder Treatment Admissions Steadily Increased In The Four Years After Medicaid Expansion.	2020
Scheeres (2020)	Changes in voluntary admission and restraint use after a comprehensive tobacco-free policy in inpatient psychiatric health facilities.	2020
Scott (2011)	Achieving and maintaining asthma control in inner-city children	2011
Shah (2011)	Evaluation of care management for the uninsured.	2011
Ferrer (2013)	Advanced primary care in San Antonio: linking practice and community strategies to improve health - PubMed	2013
Bailey (2019)	Effect of Intensive Interdisciplinary Transitional Care for High-Need, High-Cost Patients on Quality, Outcomes, and Costs: a Quasi-Experimental Study	2019
Bartholomew (2006)	Partners in school asthma management: Evaluation of a self-management program for children with asthma	2006
Bauer (2012)	Leaving before discharge from a homeless Medical Respite program: predisposing factors and impact on selected outcomes.	2012
Burns (2014)	Feasibility and evaluation of a pilot community health worker intervention to reduce hospital readmissions.	2014
Caskey (2019)	Effect of Comprehensive Care Coordination on Medicaid Expenditures Compared With Usual Care Among Children and Youth With Chronic Disease: A Randomized Clinical Trial.	2019
Castillo (2018)	Improving Depression Care for Adults With Serious Mental Illness in Underresourced Areas: community Coalitions Versus Technical Support	2018
Chisolm (2020)	A Community Development Program and Reduction in High-Cost Health Care Use.	2020
Di Masso (2001)	The clubhouse model: an outcome study on attendance, work attainment and status, and hospitalization recidivism.	2001
Finkelstein (2020)	Health Care Hotspotting - A Randomized, Controlled Trial.	2020
Gary (2009)	The Effects of a Nurse Case Manager and a Community Health Worker Team on Diabetic Control, Emergency Department Visits, and Hospitalizations Among Urban African Americans With Type 2 Diabetes Mellitus A Randomized Controlled Trial	2009
Gill (2005)	Impact of providing a medical home to the uninsured: evaluation of a statewide program.	2005
Gomez (2017)	A Cost-Benefit Analysis of a State-Funded Healthy Homes Program for Residents With Asthma: Findings From the New York State Healthy Neighborhoods Program	2017
Liu (2018)	Transition Home Plus Program Reduces Medicaid Spending and Health Care Use for High-Risk Infants Admitted to the Neonatal Intensive Care Unit for 5 or More Days.	2018
Martinez (2006)	Impact of permanent supportive housing on the use of acute care health services by homeless adults	2006
Mehta (2017)	Community Partners in Care: 6-Month Outcomes of Two Quality Improvement Depression Care Interventions in Male Participants.	2017
Rose (2016)	A Population Health Approach to Clinical Social Work with Complex Patients in Primary Care.	2016
Rubin (2019)	Association of a Targeted Population Health Management Intervention with Hospital Admissions and Bed-Days for Medicaid-Enrolled Children.	2019
Sadowski (2009)	Effect of a Housing and Case Management Program on Emergency Department Visits and Hospitalizations Among Chronically Ill Homeless Adults A Randomized Trial	2009
Szanton (2018)	Medicaid Cost Savings of a Preventive Home Visit Program for Disabled Older Adults	2018

Tinland (2020)	Effectiveness of a housing support team intervention with a recovery-oriented approach on hospital and emergency department use by homeless people with severe mental illness: a randomised controlled trial	2020
Balaban	Using a Social Worker Transition Coach to Improve Hospital-to-Home Transitions for High-Risk Nonelderly Patients.	2
Shepard (2002)	Managed care and the quality of substance abuse treatment.	2002
Smith (2004)	Improving follow-up for children with asthma after an acute emergency department visit	2004
Swaminathan (2020)	Association of Medicaid-Focused or Commercial Medicaid Managed Care Plan Type With Outpatient and Acute Care.	2020
Szilagyi (2000)	Evaluation of New York State's Child Health Plus: children who have asthma.	2000
Szilagyi (2000)	Evaluation of a state health insurance program for low-income children: Implications for State Child Health Insurance Programs	2000
Szilagyi (2006)	Improved asthma care after enrollment in the state children's health insurance program in New York	2006
Tabaei (2020)	Impact of a Telephonic Intervention to Improve Diabetes Control on Health Care Utilization and Cost for Adults in South Bronx, New York	2020
Unruh (2013)	Medicaid bed-hold policies and hospitalization of long-stay nursing home residents.	2013
Valdovinos (2020)	The association of Medicaid expansion and racial/ethnic inequities in access, treatment, and outcomes for patients with acute myocardial infarction	2020
Van Dorn (2010)	Continuing Medication and Hospitalization Outcomes After Assisted Outpatient Treatment in New York	2010
Vasan (2020)	Effects of a standardized community health worker intervention on hospitalization among disadvantaged patients with multiple chronic conditions: A pooled analysis of three clinical trials	2020
Vohra (2018)	Community health workers reduce hospitalizations and emergency department visits for low-socioeconomic urban patients with heart failure	2018
Wilson (2001)	A controlled trial of an environmental tobacco smoke reduction intervention in low-income children with asthma	2001
Woods (2012)	Community asthma initiative: evaluation of a quality improvement program for comprehensive asthma care.	2012
Wu (2009)	Asthma self-assessment in a Medicaid population.	2009
Zerzan (2007)	The demise of oregon's medically needy program: Effects of losing prescription drug coverage	2007
Zogg (2019)	Impact of Affordable Care Act Insurance Expansion on Pre-Hospital Access to Care: Changes in Adult Perforated Appendix Admission Rates after Medicaid Expansion and the Dependent Coverage Provision.	2019
Zolotor (2007)	Effectiveness of a practice-based, multimodal quality improvement intervention for gastroenteritis within a medicaid managed care network	2007

Supplementary 6: Summary of studies

	Adesanya (2005)
**Country**	Australia
**Population**	Unemployed persons
**Domain**	Health and care service-based interventions
**Intervention**	Crisis assessment and treatment services
	24h crisis assessment and treatment services for management of psychiatric crisis in the community.
	Delivered by multidisciplinary team – psychiatrist and junior medical, nursing and allied health staff
	Bi-weekly MDT discussions and on-call outreach services
**Primary outcome**	Hospital admissions
**Finding**	Post-CAT intervention, number of admissions reduced from 69 index admissions to 53. This was not statistically significant

	Burns (2007)
**Country**	Multicentre - United Kingdom, Germany, Italy, Switzerland, Netherlands, Bulgaria
**Population**	Unemployed persons
**Domain**	Population health and policy interventions
**Intervention**	Individualised jobs placement and support programmes(IPS)
	IPS workers identified patients who want to work and support them to develop goals and seek employment directly.

	IPS worker builds a network of employers willing to accept patient and supports the patient and employer during the employment (18 month follow up).
**Primary outcome**	Hospital admissions
**Finding**	The IPS group had less hospital admissions difference -11.2% (-21.5% to -0.90%) compared to those in the vocational support group.

	Castriotta (2020)
**Country**	Italy
**Population**	Deprived neighbourhoods
**Domain**	Integrative
**Intervention**	Habitat Microaree
	Support health and citizens' rights in assessment of health problems, optimisation of home healthcare to prevent institutionalisation and improper hospitalisation, promote collaboration across institutions, foster active and participatory communities
	Interventions designed depending on the needs of the microaree
**Examples of interventions**	Orientation on access to health and social services, Food bank, intergenerational activities, involvement of asylum seekers and refugees in the Microarea activity
	Self-help groups, Health promotion meetings and classes, Social breakfast and lunches to combat social isolation, vaccination campaigns,
	Assisted cohousing and support, information, orientation and support for caregivers
	Bi-weekly MDT discussions and on-call outreach services
**Primary outcome**	Hospital admissions
**Finding**	Hazard ratio of admission was 0.95 (95% CI 0.91-0.99) of the group in the intervention compared to those not in the intervention

	Downing (2019)
**Country**	United Kingdom
**Population**	Deprived neighbourhood
**Domain**	Integrative
**Intervention**	Knowsley CVD Service (KCVDS)
	Consultant-led multidisciplinary clinic provided from primary care centres offering same day assessment and testing in clinic and at home.
	Direct access to diagnostics services
	Community heart failure clinic, cardiovascular rehabilitation and stroke rehabilitation services.
	Delivered by a heart failure nursing team, cardiac rehabilitation and medical consultants.
**Primary outcome**	Hospital admissions
**Finding**	66 fewer cardiovascular disease related admissions per 100 000 population per year – DiD estimator -65.56 (95% CI -108.98 to -22.13) p=0.003

	Forget (2011)
**Country**	Canada
**Population**	Low income population group
**Domain**	Population health and policy interventions
**Intervention**	MINCOME – Manitoba Basic Annual Income Experiment
	Guaranteed annual income or minimum cash benefit for lower-income households depending on family size. The amount was reduced for every dollar they earned by working.
**Primary outcome**	Hospital admissions
**Finding**	Found an 8.5% reduction in hospitalisation rate for participants relative to control, particularly for accidents and injuries

	Gazey (2019)
**Country**	Australia

**Population**	People experiencing homelessness
**Domain**	Integrative
**Intervention**	The Cottage a medical respite centre
	Six-bed respite facility for people experiencing homelessness or at risk of homelessness to receive acute nursing and support post-discharge
	Staffed by nursing and personal care staff
	Links clients with other services and assist in obtaining more permanent accommodation
**Primary outcome**	Hospital admissions
**Finding**	Proportion of clients admitted to hospital reduced by 18% after receiving care from The Cottage

	Horwitz (2021)
**Country**	Israel
**Population**	Low-income population
**Domain**	Integrative
**Intervention**	Health education on asthma
	Two coaching sessions, one in the hospital and one at home. During the home visit, a nurse checked for environmental factors as well as inhaler technique and adherence
	Asthma Control Test (ACT) questionnaire across the intervention to monitor and evaluate impact
	Delivered by multidisciplinary team – pulmonologist, asthma nurse
**Primary outcome**	Hospital admissions
**Finding**	No difference in hospital admissions between intervention and control groups

	Hwang (2011)
**Country**	Canada
**Population**	People experiencing homelessness
**Domain**	Integrative
**Intervention**	Supportive housing for people experiencing homelessness and vulnerably housed individuals
	Access to drop-in centre offering meals, and outreach services as well as medical and dental clinic providing free services and onsite mental health and community support
	Individuals receive rental subsidies and paid rent-geared-to-income
	Support workers available to support residents with mental illness, transition into the housing program and providing ongoing health with living skills, counselling and advocacy
**Primary outcome**	Hospital admissions
**Finding**	No difference in hospital admission rate between intervention and usual care groups
**Secondary outcomes**	Reduced alcohol and drug use

	Jackson (2011)
**Country**	New Zealand
**Population**	Low-income population
**Domain**	Integrative
**Intervention**	Health education on health risks, referral to local health providers
	Housing improvements: insulation, heating, ventilation modifications, transferring families to other houses to address overcrowding
**Primary outcome**	Hospital admissions
**Finding**	Combined interventions to improve health and social service access and housing conditions is associated with a reduced rate of acute admissions in those aged 0-34 years HR 0.73 (0.58-0.91)

	Kackin and Kahraman (2020)
**Country**	Turkey

**Population**	Low income population
**Domain**	Health and care service-based interventions
**Intervention**	Health education program
	Group training sessions on discharge regarding asthma. The sessions used videos, presentation, and questions and answers.
	Follow up with home monitoring three months after discharge to assess inhaler technique and troubleshoot questions
	Bi-weekly MDT discussions and on-call outreach services
**Primary outcome**	Hospital admissions
**Finding**	Number of children with >10 hospital admission per 3 months reduced from 31.2% to 7%

	Kim and Shon (2018)
**Country**	South Korea
**Population**	Low income population
**Domain**	Population health and policy interventions
**Intervention**	Medical aid health program
	Government's medical benefit program – a public assistance scheme to secure the minimum livelihood for low-income households and to assist self-help by providing medical services
	Type 1 Medical Aid, those unable to work aged <18years or >65years and intangible cultural assess e.g. people injured or died whilst saving other people
	Type 2 Medical aid those aged between 18-65 able to work. The two types offer different medical out-of-pocket expense plan, which is relative low compared to National Health Insurance
**Primary outcome**	Hospital admissions
**Finding**	Those in the Medical Aid scheme had an increased in hospitalization rate compared to those in the National Health Insurance program

	Lichtl (2019)
**Country**	Germany
**Population**	Low income population
**Domain**	Health and care service-based interventions
**Intervention**	Walk-in clinic in a asylum seeker reception centre area
	Consultations for general medicine, gynaecology, paediatrics, tropical medicine, psychiatry – both treatment of acute illness and preventive medical check=ups
**Primary outcome**	Hospital admissions
**Finding**	Post intervention, the number of admissions was reduced among asylum seeker (incidence-- rate ratios (IRR)=0.80 (0.65 to 1.00), p=0.054), but the effect was attenuated after adjustment for time trends.

	Lopez Cabezas (2006)
**Country**	Spain
**Population**	Low income population
**Domain**	Health and care service-based interventions
**Intervention**	Health education program on heart failure
	Personal interview on the day of hospital discharge to provide information on the disease, diet education and information on drug therapy
	Telephone contact of the pharmacist provided for the patient and their relative
	Monthly telephone contacts to strengthen the intervention and sole any doubts that could have arisen
	Delivered by a pharmacist
**Primary outcome**	Hospital admissions
**Finding**	Hospital admission reduced in intervention group (0.13 per patient) compared to control group (0.41 per patient), p=0.034

	Rodgers (2018)
**Country**	United Kingdom
**Population**	Low income population
**Domain**	Population health and policy interventions
**Intervention**	Housing quality improvement
	Internal and external housing improvements, including wall and loft insulation, windows and door, heating system upgrades, new kitchens and bathrooms, garden path safety improvement and electrical system upgrades to meet a specific standard
**Primary outcome**	Hospital admissions
**Finding**	A reduction in hospital admission was associated with housing interventions across all ages, particularly for improvements in windows, doors and wall insulation

	Tinland (2020)
**Country**	France
**Population**	People experiencing homelessness
**Domain**	Integrative
**Intervention**	Housing First: participants offered scattered housing
	Subsidies provided to ensure participants paid no more than 30% of their income on rent
	Assertive Community Treatment (ACT) model with a recovery-oriented approach delivered by multidisciplinary team – social worker, nurse, doctor, psychiatrist and peer worker
	One weekly visit at home or city
**Primary outcome**	Hospital admission
**Finding**	Relative risk 0.96 (0.76-1.21) p=0.449

	Goldzahl (2022)
**Country**	United Kingdom
**Population**	Low income population
**Domain**	Integrative
**Intervention**	Multi-Disciplinary Groups (MDG)meeting
	Multi-professionals sharing information meetings, discussing and care planning for high risk patient cases. Delivered an integrated contact centre providing advice to navigate health and social care services for high risk patients
	MGDs increased support and support to community services
	MDG composed of general practitioner, mental health professional, social worker, geriatrician and nurses
**Primary outcome**	Hospital admissions
**Finding**	Hospital admission remained unchanged (−0.044 (SE 0.049))

	Malden (2023)
**Country**	United Kingdom
**Population**	People experiencing homelessness
**Domain**	Integrative
**Intervention**	Hospital-in-reach programme
	Multidisciplinary, multicomponent intervention involving clinical and non-clinical healthcare staff based across secondary care, primary care, and community sector
	Upon identification through a purpose-built clinical decision support algorithm, patients were assessed into one of three interventions depending on their needs
	(1) One touch: One type of support or treatment, e.g. securing accommodation
	(2) Light touch: receiving more than one specific intervention, e.g. accommodation support along with emergency provisions and support referral
	(3) Casework: Most comprehensive, comprised more than one intervention and ongoing support after discharge in the community

**Primary outcome**	Hospital admissions
**Finding**	Hospital admission were reduced by -68.7% from baseline to 12 months follow up for all interventions, this was statistically significant p<0.01

	Norris (2023)
**Country**	New Zealand
**Population**	Low income
**Domain**	Population health and policy interventions
**Intervention**	Exemption from prescription co-payments
	Low income group exempt from co-payments for all medicines they receive
**Primary outcome**	Hospital admissions
**Finding**	Mean number of hospital admission was 0.77 (SD 1.88) for those in the intervention group and 0.97 (SD 1.79) in the control group, p-value 0.07

	Ress (2024)
**Country**	Germany
**Population**	Low income
**Domain**	Integrative
**Intervention**	Network of health, social and community care providers
	Network meeting aim to improve collaboration between providers and health professionals
	Community health advice and navigation service available in multiple languages provided by community health nurses for patients to receive advice and education
	Institutions can refer participants for social prescribing
**Primary outcome**	Hospital admissions
**Finding**	Difference in difference increase by 0.016 SE 0.003 p-value <0.001

	Salvalaggio (2022)
**Country**	Canada
**Population**	Low income population
**Domain**	Health and care service-based interventions
**Intervention**	Bridging intervention to promote care continuity during hospital community care transition
	Management of intoxication, withdrawal and/or acute pain, overdose prevention intervention referral to addiction treatment and recovery program, screening for sexually transmitted infection
	Delivered by rotating physicians with addiction medicine expertise, nurse practitioner, social worker, addiction counsellor and peer support worker
	Intensity of the components were tailored to patient needs and preferences
**Primary outcome**	Hospital admissions
**Finding**	Adjusted OR 0.89 (0.55,1.45) for reduction in Inpatient Admissions, p-value 0.633
